# Nitrogen Incorporation in Potassic and Micro- and Meso-Porous Minerals: Potential Biogeochemical Records and Targets for Mars Sampling

**DOI:** 10.1089/ast.2021.0158

**Published:** 2022-10-31

**Authors:** Matthew P. Nikitczuk, Gray E. Bebout, Charles A. Geiger, Tsutomu Ota, Takuya Kunihiro, John F. Mustard, Sæmundur A. Halldórsson, Eizo Nakamura

**Affiliations:** ^1^ Department of Earth and Environmental Sciences, Lehigh University, Bethlehem, Pennsylvania, USA.; ^2^ Pheasant Memorial Laboratory for Geochemistry and Cosmochemistry, Institute for Planetary Materials, Okayama University, Misasa, Japan.; ^3^ Universität Salzburg, Fachbereich Chemie und Physik der Materialien, Salzburg, Austria.; ^4^ Department of Earth, Environmental and Planetary Sciences, Brown University, Providence, Rhode Island, USA.; ^5^ Nordic Volcanological Center, Institute of Earth Sciences, University of Iceland, Reykjavík, Iceland.

**Keywords:** Stable isotopes, Mars, Biogeochemistry, Planetary habitability and biosignatures, Silicates

## Abstract

We measured the N concentrations and isotopic compositions of 44 samples of terrestrial potassic and micro- and meso-porous minerals and a small number of whole-rocks to determine the extent to which N is incorporated and stored during weathering and low-temperature hydrothermal alteration in Mars surface/near-surface environments. The selection of these minerals and other materials was partly guided by the study of altered volcanic glass from Antarctica and Iceland, in which the incorporation of N as NH_4_^+^ in phyllosilicates is indicated by correlated concentrations of N and the LILEs (*i.e.*, K, Ba, Rb, Cs), with scatter likely related to the presence of exchanged, occluded/trapped, or encapsulated organic/inorganic N occurring within structural cavities (*e.g.*, in zeolites). The phyllosilicates, zeolites, and sulfates analyzed in this study contain between 0 and 99,120 ppm N and have δ^15^N_air_ values of −34‰ to +65‰. Most of these minerals, and the few siliceous hydrothermal deposits that were analyzed, have δ^15^N consistent with the incorporation of biologically processed N during low-temperature hydrothermal or weathering processes. Secondary ion mass spectrometry on altered hyaloclastites demonstrates the residency of N in smectites and zeolites, and silica. We suggest that geological materials known on Earth to incorporate and store N and known to be abundant at, or near, the surface of Mars should be considered targets for upcoming Mars sample return with the intent to identify any signs of ancient or modern life.

## 1. Introduction

Nitrogen is a key element for understanding the evolution of terrestrial planets (see Canfield *et al.*, 2010; articles in Bebout *et al.*, 2013a). The mass fractions and isotope compositions of nitrogen (N) in atmospheric and geologic reservoirs can provide useful information for determining the nature of precursor materials, the long-term evolution of atmospheres (*e.g.*, volatile outgassing), habitable past environmental conditions, and possibly (bio)geochemical cycling processes (Boyd, 2001; Zerkle and Mikhail, 2017).

Reactive fixed N species such as NH_3_, NH_4_^+^, and NO_3_^−^ are particularly important in the origin and maintenance of life on Earth. Since fixed N is required for assimilation into DNA, RNA, and protein precursor biomolecules, such as amino acids, and because microbes play a crucial role in the N_2_ fixation process, it is a compelling potential tracer for astro- and exobiological processes (see Capone *et al.*, 2006).

Viking lander and Mars Science Laboratory (MSL) measurements reveal that Mars' atmosphere contains a significantly lower concentration of N_2_ (∼2.7% by volume and ∼0.15–0.2 mbar) compared with that of Earth, and it is isotopically heavier (*i.e.*, δ^15^N = +572 ± 82‰ with ^15^N/^14^N normalized to the present ratio of that in Earth's atmosphere; Mahaffy *et al.*, 2013; Wong *et al.*, 2013). Mars' primordial volatile inventory, however, is estimated to have been rich in N_2_ corresponding to 3–300 mbar (McKay and Stoker, 1989), in addition to containing CO_2_, H_2_O, and other volatiles (see modeling by Kurokawa *et al.*, 2018).

The ^15^N enrichment of Mars' atmosphere may have resulted from progressive fractionated loss to space through escape processes (Brinkmann, 1971; McElroy, 1972; McElroy *et al.*, 1976; Fox and Dalgarno, 1983), solar wind stripping (Jakosky *et al.*, 1994), or impact erosion. An appreciable quantity of initially atmospheric N could, however, be bound within surface/near surface materials such as regolith-buried nitrates and nitrites, which typically occur in very dry desert soils on Earth (Mancinelli, 1996; Mancinelli and Banin, 2003; Stern *et al.*, 2015) or as structurally stabilized NH_4_^+^ in the crystal structures of potassic minerals such as certain phyllosilicates (Mancinelli, 1996; Bishop *et al.*, 2002; Mancinelli and Banin, 2003).

NH_4_^+^ has not yet been identified on Mars. However, the recent detections of nitrates in eolian sediments from Gale Crater (Stern *et al.*, 2015, 2018), martian meteorites (*e.g.*, EETA79001, Tissint), and N-bearing organic compounds in minerals of Tissint and ALH 84001 meteorites believed to be indigenous to Mars (Kounaves *et al.*, 2014; Jaramillo *et al.*, 2019; Koike *et al.*, 2020) indicate that atmospheric gases (*e.g.*, H_2_O, CO_2,_ N_2_) may be exchanged with, and stored within, martian surface and near-surface materials. The specific minerals and other phases that could house various N-bearing species and potentially preserve biogeochemical records have remained uncertain (see Mancinelli and Banin, 2003).

Orbital spectroscopic, *in situ* lander and rover exploration, and theoretical mineralogical considerations indicate that most of Mars' upper crust is composed of basaltic volcanic rocks and sediments. The latter contain various authigenic minerals consistent with low temperature hydrothermal weathering and diagenetic processes (Gregg and Williams, 1996; Bandfield *et al.*, 2000; Poulet *et al.*, 2007; Squyres *et al.*, 2007; Ehlmann and Edwards, 2014; Schmidt *et al.*, 2014; Vaniman *et al.*, 2014). Amorphous silicates have also been detected (Michalski *et al.*, 2005; Chevrier *et al.*, 2007; Horgan and Bell, 2012; De Vet *et al.*, 2014; Grotzinger *et al.*, 2014; Vaniman *et al.*, 2014; Bristow *et al.*, 2018), and these appear to represent variable deposits of altered volcanic or impact-derived glasses.

Fe-Mg phyllosilicates (*e.g.*, nontronite-saponite) are typical of basalt weathering and common, but more locally dominant Al-phyllosilicates (*e.g.*, kaolinite, montmorillonite) (Poulet *et al.*, 2005; Bibring *et al.*, 2006; Mustard *et al.*, 2008; Carter *et al.*, 2013; Sun and Milliken, 2015) and various other hydrous minerals are present as well. These include sulfosalts (sulfates) such as kieserite (Arvidson *et al.*, 2005), gypsum and polyhydrated sulfates (Gendrin *et al.*, 2005), jarosite (Squyres *et al.*, 2004a, 2004b; Milliken *et al.*, 2008), and alunite (Swayze *et al.*, 2008).

Also observed is opaline silica, which occurs as rock coatings (Kraft *et al.*, 2003; Michalski *et al.*, 2005) or aqueous and hydrothermal/fumarolic deposits (Arvidson *et al.*, 2008; Squyres *et al.*, 2008; Ruff and Farmer, 2016), carbonates (Ehlmann *et al.*, 2008; Morris *et al.*, 2010; Niles *et al.*, 2013), and zeolites such as analcime related to impact-induced hydrothermal systems (Ehlmann *et al.*, 2009; Carrozzo *et al.*, 2017). Occurrences of the two zeolites chabazite and clinoptilolite have also been suggested (Mousis *et al.*, 2016) based on spectroscopic observations (Ruff, 2004; Michalski *et al.*, 2005).

However, because the chemical signatures of many clays and zeolites can be similar and there are differences between the spatial resolution and detection limits of orbital versus *in situ* measurements (Sætre *et al.*, 2019), the amount of zeolites on the Mars surface may be underestimated. This could be important because, like phyllosilicates, zeolites typically occur as glass alteration products (Stroncik and Schmincke, 2002; see Nikitczuk *et al.*, 2022a). Moreover, in pyroclastic and impact deposits, they have the potential to be sorbents, UV-shields for organic compounds, or reactive catalysts that could serve as records of martian atmospheric and hydrosphere chemistry (Ming and Gooding, 1988).

Considering these various issues, we undertook an investigation to measure the N concentrations and isotope compositions of various authigenic minerals and phases known on Earth to be alteration products of basalt and that have been identified, or could be present, on the martian surface. Many of the phases chosen for study have also been identified in altered hyaloclastites sampled from Iceland, Oregon, Antarctica, and elsewhere (see discussion by Nikitczuk *et al.*, 2022a). The N concentrations and isotope compositions presented for the various phases studied here were compared with those of terrestrial altered basalts and hyaloclastites and both pristine and altered mid-ocean ridge basalts (MORB) and ocean-island basalts (OIB).

In this framework, this study constitutes an in-depth survey of the N retentivity in various Mars-analog phases and minerals. The results serve to build a data base for understanding the potential of these materials to preserve biogeochemical and environmental processes and records on planets and, here, Mars. Some of these phases, particularly the zeolites, Fe-rich smectite (*i.e.*, nontronite, a typical alteration product of basalt), jarosite (hydrated K sulfates), and apophyllite (in which K^+^ and NH_4_^+^ can reside), were considered for the first time, in the present study, with regard to their N concentrations and isotope compositions.

We analyzed illite (*i.e.*, a K-rich clay mineral), for which many studies have documented elevated N concentrations (see Bobos and Williams, 2017) in a variety of settings (hydrothermal deposits, authigenic phases in clastic rocks at depth in sedimentary basins). Finally, and, in addition, we measured several other materials identified on the martian surface, such as amorphous silica (opal) or cristobalite (quartz polymorph). These are of interest because of their enhanced potential to incorporate N and preserve textural and biogeochemical evidence of putative martian microbial life (see the recent discussions by Ruff and Farmer, 2016; Steller *et al.*, 2019; Ruff *et al.*, 2020).

## 2. Materials and Methods

### 2.1. Various Mars surface/near-surface analog materials for nitrogen analyses

The suite of samples comprises individual mineral specimens separately sampled from various formation environments. Most of the phyllosilicates and zeolites analyzed in this study were formed as secondary or late minerals in diagenetic or in low-temperature processes such as in some hydrothermal systems. Basaltic and silicic volcanics and intrusives were the primary source rocks, and the secondary mineral phases occur as either alteration products of primary high-temperature glass or minerals.

Other materials occur as pore-filling cements and in amygdales or vugs. Many other phases have been either identified spectroscopically on the martian surface via satellite observations (see the review by Ehlmann *et al.*, 2011) or detected in different rover missions (*e.g.*, Squyres *et al.*, 2004a, 2004b, 2008; Arvidson *et al.*, 2008; Schmidt *et al.*, 2009; see the discussion in Section 4). Some phyllosilicates formed in glacio- fluvio-lacustrine sediments (CLA005, 84754 Illite), whereas silica deposits include hot spring sinters (ICE003, MAF700) and sulfate deposits include evaporitic lacustrine (SPT-158 thenardite) and acid-mine drainage (32918 jarosite) conditions.

We compared our data with the whole-rock data of Nikitczuk *et al.* (2022a) for hyaloclastite tuffs and breccias from the Eastern and Western Volcanic rift zones, Iceland, and the Carapace Nunatak, South Victoria Land, Antarctica, respectively. Although the Carapace sandstone, studied by Cannon *et al.* (2015), is related to the Antarctic hyaloclastites in that it contains altered glassy basaltic-andesitic debris, it is a volcaniclastic sandstone composed of rock fragments, quartz, feldspars, micas, and a mostly zeolite cement.

### 2.2. Analysis of nitrogen concentrations and isotopic compositions

We employed sealed-tube-combustion and carrier-gas methods using a system that couples an all metal, low-blank vacuum extraction and cryo-purification line built at Lehigh University with a Finnigan Gas Bench II carrier gas system (see the description of methods by Bebout *et al.*, 2007). For all measurements, 8–252 mg of crushed material were loaded into 6 mm (o.d.) quartz tubes, along with 1 g of Cu_x_O_x_ reagent.

The tubes were then evacuated for 24 h on a glass vacuum line, with intermittent heating to ∼100°C to remove adsorbed atmospheric N. They were then sealed under vacuum and heated to 1050 °C in a programmable muffle furnace. Tubes were cracked onto the high vacuum metal extraction line, and cryogenically purified molecular N (N_2_) was then transferred into a Finnigan Gas Bench II continuous-flow interface where it was entrained in a He stream and conveyed into a Finnigan MAT 252 isotope ratio mass spectrometer. The N isotope compositions are reported in conventional delta notation (Eq. 1) as per mil (‰) with respect to terrestrial atmospheric N_2_ (and reported as δ^15^N_air_) where:


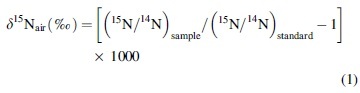


Sealed tubes containing only the Cu/CuO_x_ regent (reagent blanks) were regularly analyzed during analytical runs to monitor the contribution of the combustion reagent and the extraction vacuum system to measured N_2_. To determine N concentrations, measured N peak area was interpolated to an internal silicate standard-calibrated relationship between N peak area and N concentrations. Nitrogen concentrations are reported as ppm (μg/g). The analytical uncertainties for N concentrations are usually <5%. Uncertainties for δ^15^N values are 0.15‰ (1σ) for samples with >5 μg/g N, and 0.6‰ (1σ) for samples with 1–5 μg/g N.

### 2.3. Mapping of nitrogen and carbon (and major element) concentrations

To demonstrate the residency of N (and C) in many of the phases studied here, mapping of ^12^C-, ^13^C-, ^12^C^14^N, ^12^C^15^N, ^28^Si- and ^35^Cl- was conducted on palagonitized volcanic glass via secondary ion mass spectrometry (SIMS) using a Cameca IMS1280-HR at the Pheasant Memorial Laboratory, Institute for Planetary Materials, Japan. Polished 2.5 cm round thin sections were Au-coated (200 nm) with a JEOL JFC-1500 ion sputtering device. A primary Cs^+^ beam impacted the sample surface at −20 keV. For mapping of 80 × 80 μm areas, the primary beam current was set to 3 pA with a 1 nA 180 s pre-sputtering (100 μm^2^) and 30 s automatic mass calibration period. A contrast aperture of 400 μm was used, and the energy band pass width was also set to −10 to 40 eV. The entrance and exit slit widths were set to 50 and 150 μm, respectively. The resultant mass resolution (m/Δm) of ∼8000 could separate interferences of ^12^CH^−^ and ^13^C^14^N^−^. Each run consisted of three to four cycles.

X-ray mapping of the major elements was conducted by an electron probe microanalyzer in wavelength-dispersive mode with a JEOL JXA-8800 and a JEOL JXA-8530F, and both were also housed at the Pheasant Memorial Laboratory, Institute for Planetary Materials, Okayama University, Japan. The mapping was performed at modified conditions using 15 kV accelerating voltage, working distance of 11 mm, 200-nA probe currents, and 10–20 ms dwell time per 1–3-μm pixel, depending on the target dimension. Natural silicates and synthetic oxides on ASTIMEX MINM25–53 were used as standards.

## 3. Results

### 3.1. Nitrogen concentrations and isotopic compositions of potassic and micro- and meso-porous minerals

**FIG. 1.**
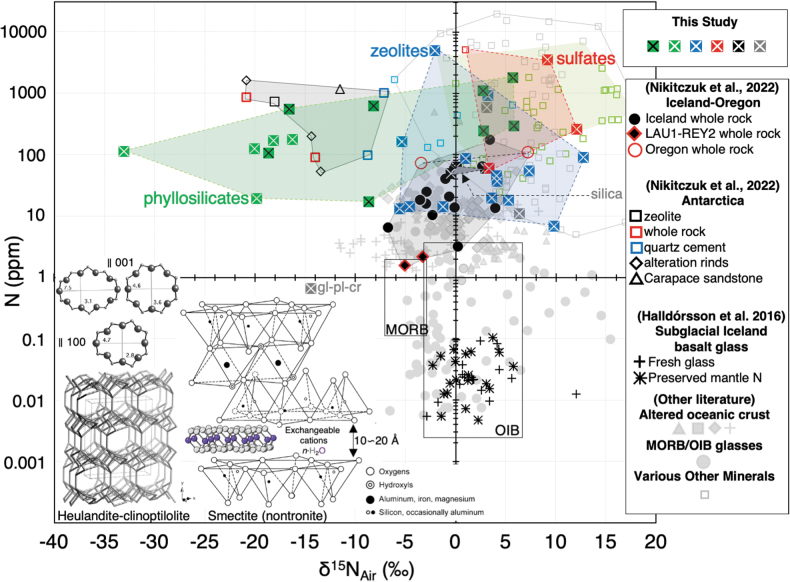
Nitrogen isotope compositions as a function of N concentration in potassic and micro- and mesoporous Mars-analog minerals from globally distributed locations. For reference, also plotted are previously published data for whole rock and physical separates of alteration rinds and cements from Antarctic basaltic andesitic flow foot breccias and Iceland and Oregon basaltic tuffs (presented in Nikitczuk *et al.*, 2022a). See Table 1 for a list of minerals and N data for this study plotted here and Nikitczuk *et al.* (2022a, 2022b) for hyaloclastite N data. Data point labeled as gl-pl-cr represents a mixture of glass, plagioclase, and cristobalite. Also plotted from the literature and outlined in the gray dashed line are buddingtonite (NH_4_^+^-rich feldspar), alunite (sulfate), muscovite/biotite (phyllosilicate-micas), illite-smectite/chlorite, melanophlogite (silica clathrasil), and beryl/cordierite (cyclosilicates) from modern and fossil hot springs, low temperature to hydrothermal and metamorphic sedimentary environments (Krohn *et al.*, 1993; Boyd, 1997; Papineau *et al.*, 2005; Svensen *et al.* 2008; Lazzeri, 2012, Lazzeri *et al.*, 2017; Bebout *et al.*, 2016; Bobos and Williams, 2017). The green area outlining the phyllosilicates also includes micas and illite-smectite-chlorite from the literature (no dashed outline), which extends to the right side of the plot, and shows that this mineral group has the widest range in isotope compositions. Phyllosilicate data points from this study with white Xs represent smectites. The red area outlining the sulfates from this study also includes one sample of alunite from the literature (Krohn *et al.*, 1993). The nitrogen concentrations (ppm) of minerals from literature were converted from weight percent (NH_4_)_2_O or N, or from parts per million NH_4_^+^. General ranges for MORB and OIB are outlined in boxes at the lower right (Marty and Zimmerman, 1999; Marty and Dauphas, 2003; from Cartigny and Marty, 2013). Other data for basalts are for fresh subglacial Iceland basaltic glasses (from Halldórsson *et al.*, 2016; values converted from μcm^3^ STP/g to μg/g), with those representing unmodified mantle signatures based on ^40^Ar/^36^Ar and ^4^He/^40^Ar ratios indicated, and fresh MORB and OIB glasses (Exley *et al.*, 1986/1987; Sakai *et al.*, 1984; Marty and Humbert, 1997; Cartigny *et al.*, 2001), and for altered oceanic crust (Busigny *et al.*, 2005; Li *et al.*, 2007; Bebout *et al.*, 2018). At the bottom left are crystal structural schematic diagrams for the framework zeolite heulandite-clinoptilolite (left; from Database of Zeolite Structures https://www.iza-structure.org) and the phyllosilicate (smectite) nontronite (modified from Murray, 2006) showing the channel openings and interlayer space, respectively, in which cations and molecules such as K^+^, NH_4_^+^, H_2_O, or organics can potentially be incorporated and retained either post-formation through exchange/diffusion processes, or occluded/trapped during formation (also see Kolesov and Geiger, 2006; Geiger and Dachs, 2009; Geiger *et al.*, 2010). Dimensions of pore openings and interlayer space are given in Å. For heulandite-clinoptilolite, the maximum diameter of a spherical molecule that can be included within the structure is 5.97Å and that which can diffuse through the structure is 3.67Å. MORB, mid-ocean ridge basalts; N, nitrogen; OIB, ocean island basalts; STP, standard temperature and pressure.

The measured N concentrations and isotopic compositions of the various minerals and phases are presented in Table 1 and Fig. 1. There is a large range in both N concentrations, from 0 to 99,120 ppm, and in δ^15^N, from −34.0‰ to +65.4‰. The phyllosilicates show the widest range in N concentration from 17 to 99,120 ppm (with all but three samples having <2000) followed by the zeolites with 0.5 to 4827 ppm, sulfates with 59 to 3460 ppm, and lastly silica with 48 to 62 ppm (δ^15^N = −1.4‰ to +2.1‰). The highest N concentration is from a 2:1 layer clay mineral, whereas the lowest concentrations (<0.1–0 ppm) are from the apophyllite samples and two zeolites, heulandite (8fl2–003), and mesolite (M-1).

**Table 1.** Nitrogen Concentrations and Isotope Compositions of Potassic and Micro- and Meso-Porous Mars-Analog Minerals (and Siliceous Deposits)

Sample	Mineral	Group	Ideal formula	Locality	Sample size (mg)	[N] (ppm)	δ^15^N (‰)
RC-214^a^	Apophyllite	p	KCa_4_Si_8_O_20_(F,OH)·8H_2_O	Poona, Bombay, India	9.6	1754	+4.8
RC-414	Apophyllite	p	KCa_4_Si_8_O_20_(F,OH)·8H_2_O	Loudon County, VA, USA	9.6	0.0	—
Apophyllite (Jalg.) India)	Apophyllite	p	KCa_4_Si_8_O_20_(F,OH)·8H_2_O	Jalgoan, India	11.4	0.0	—
RC-298	Fluorapophyllite	p	KCa_4_Si_8_O_2_0(F,OH)·8H_2_O	Nashik, India	10.6	1.6	+47.0
104463	Fluorapophyllite-K	p	KCa_4_Si_8_O_2_0(F,OH)·8H_2_O	Pune, India	9.3	0.0	—
CLA005	2:1 clay	p	Al_2_Si_4_O_10_(OH)_2_	Pembina Valley, MB, Canada	52.6	99120	−1.0
HAL001^a^	Halloysite	p	Al_2_(Si_2_O_5_)(OH)_4_	Tintic District, UT, USA	99.5	106	−19.6
RC-4034^a^	Halloysite	p	Al_2_(Si_2_O_5_)(OH)_4_	Grubb Mine, VA, USA	55.3	540	−17.5
RC-4035^a^	Halloysite	p	Al_2_(Si_2_O_5_)(OH)_4_	Liege, Belgium	22.8	239	+1.8
84754 Illite^a^	Illite	p	(K,H_3_O)(Al,Mg,Fe)_2_(Si,Al)_4_O_10_[(OH)_2_,(H_2_O)]	Temple Mountain, UT, USA	21.4	289	+4.9
KAO104	Kaolinite	p	Al_2_Si_2_O_5_(OH)_4_	Warren County, GA, USA	50.6	17	−9.6
LU-3990	Lithomarge	p	Al_2_Si_2_O_5_(OH)_4_	Friedensville, PA, USA	57.5	1086	+1.8
RC-3982^a^	Lithomarge	p	Al_2_Si_2_O_5_(OH)_4_	Rochlitz, Saxony, Germany	53.9	616	−9.1
17898^a^	Montmorillonite	p	(Na,Ca)_0.3_(Al,Mg)_2_Si_4_O_10_(OH)_2_•n(H_2_O)	Kiakhta (Khyagt), Russia	94.9	123	−21.0
LU-3831	Nontronite	p	Na_0.3_Fe_2_(Si,Al)_4_O_10_(OH)_2_·nH_2_O	Saucon Valley, PA, USA	80.4	172	−17.2
LU-3830^a^	Nontronite	p	Na_0.3_Fe_2_(Si,Al)_4_O_10_(OH)_2_·nH_2_O	Saucon Valley, PA, USA	64.9	167	−19.1
26219	Nontronite	p	Na_0.3_Fe_2_(Si,Al)_4_O_10_(OH)_2_·nH_2_O	Garfield, WA, USA	83.4	19	−20.8
111976^a^	Saponite	p	Ca_0.3_(Mg,Fe)_3_((Si,Al)_4_O_10_)(OH)_2_·nH_2_O	Hector, CA, USA	102.8	110	−34.0
HAW049	Palagonite	pal	—	Mauna Kea, HI, USA	100.7	577	+2.2
40065	Analcime	z	Na(AlSi_2_O_6_)•H_2_O	Mont Saint Hilaire, QB, Canada	39.7	6.9	+8.9
Armenite	Armenite	z	BaCa_2_Al_6_Si_9_O_30_ · 2H_2_O	Wasenalp, Valais, Switzerland	39.8	19	+2.6
88538	Chabazite	z	(K_2_,Ca,Na_2_,Mg,Sr)[Al_2_Si_4_O_12_]•6(H_2_O)	Cape Blomidon, NS, Canada	42.9	14	−5.6
Ch-2	Chabazite	z	(K_2_,Ca,Na_2_,Mg,Sr)[Al_2_Si_4_O_12_]•6(H_2_O)	Faroe Islands, Denmark	43.7	85	0.0
Chabazite (Csodi)	Chabazite	z	(K_2_,Ca,Na_2_,Mg,Sr)[Al_2_Si_4_O_12_]•6(H_2_O)	Csodi Hill, Hungary	40.9	13	−6.6
31861^a^	Clinoptilolite	z	(Ca,K,Na)_3–6_(Si_30_Al_6_)O_72_·20H_2_O	Canadon Hondo, Argentina	53.0	161	−6.3
8f10-017	Heulandite	z	(K,Na,Ca)_2-3_Al_3_(Al,Si)_2_Si_13_O_36_•12(H_2_O)	Fassatal, South Tirol, Italy	46.2	54	+6.4
47728	Heulandite	z	(K,Na,Ca)_2-3_Al_3_(Al,Si)_2_Si_13_O_36_•12(H_2_O)	Coonabarabran, Australia	86.5	14	−2.2
8f12-003	Heulandite	z	(K,Na,Ca)_2-3_Al_3_(Al,Si)_2_Si_13_O_36_•12(H_2_O)	Iceland	41.2	0	−
*M*-1	Mesolite	z	Na_2_Ca_2_Al_6_Si_9_O_30_•8(H_2_O)	Faroe Islands, Denmark	41.7	0	−
Mesolite (Pune)^a^	Mesolite	z	Na_2_Ca_2_Al_6_Si_9_O_30_•8(H_2_O)	Pune, MH, India	41.3	909	2.3
MM-6759^a^	Mordenite	z	(Ca,Na_2_,K_2_)(Al_2_Si_10_)O_24_•7(H_2_O)	New Water Mts., AZ, USA	21.7	4827	−3.0
Ph-1^a^	Phillipsite	z	(K,Na,Ca)_1-2_(Si,Al)_8_O_16_•6(H_2_O)	Ouro Preto, Brazil	40.2	90	+11.8
Scolecite-xx	Scolecite	z	CaAl_2_Si_3_O_10_•3(H_2_O)	Chalisgaon, India	60.9	18	+4.3
8f8-22	Scolecite	z	CaAl_2_Si_3_O_10_•3(H_2_O)		40.6	0.5	+65.4
ST-1	Stellerite	z	CaAl_2_Si_3_O_10_•3(H_2_O)	Grant County, OR, USA	39.6	36	+3.2
8f13/016	Yugawaralite	z	CaAl_2_Si_6_O_16_•4(H_2_O)	Bombay, India	40.9	45	+3.1
81233^a^	Opal	Si	SiO_2_•n(H_2_O)	Nye County, NV, USA	103.5	62	−0.9
ICE003^a^	Amorphous silica	Si	SiO_2_	Geysir, Iceland	100.9	60	+2.1
MAF700 (silica)	Silica (quartz+calcite)	si, c	SiO_2_, CaCO_3_	Mafeking, MB, Canada	84.5	48	−1.4
25821	Alunite	su	KAl_3_(SO_4_)_2_(OH)_6_	Carrickalinga Head, Australia	25.6	59	+2.4
32917^a^	Jarosite	su	KFe^3+^_3_(SO_4_)_2_(OH)_6_	San Jose, Oruro, Bolivia	17.9	3460	+8.2
SPT-158.r	Thenardite	su	Na_2_(SO_4_)	Chaplin, SK, Canada	53.1	257	+11.1
86621	Leucite	f, si	KAl(Si_2_O_6_)	Mt. Vesuvius, Naples, Italy	105.0	11	+5.4
MSH ASH	Glass plagioclase cristobalite	ash, f, si	(Na,Ca)(Si,Al)_4_O_8_, SiO_2_	Mt. St. Helens, WA, USA	10.0	0.7	−15.4

Sample sources: American Museum of Natural History, Canadian Space Agency Planetary Analogue Suite, Lehigh University, Universität Salzburg.

^a^ Indicates N concentration and δ^15^N values that are averages of two separate measurements from samples that required splitting within the extraction line to avoid detector oversaturation.

c = carbonate; f = feldspar; p = phyllosilicate; si = silica; su = sulfate; z = zeolite.

The phyllosilicate, zeolite, and sulfate groups have at least one specimen with N concentrations >1000 ppm. The most negative and the most positive δ^15^N values are for the smectite clay saponite (sample 111976) and the zeolite scolecite (sample 8f8–22), respectively. Smectite clays (saponite, nontronite, montmorillonite) have 19–172 ppm N and δ^15^N of −34‰ to −17.2‰. Similarly, two of the halloysites have N concentrations of 106 ppm and 540 ppm and δ^15^N values of −17.5‰ and −19.6‰. Illite (sample 84754) and fluorapophyllite (sample RC-298) have the most positive δ^15^N values of the phyllosilicates (+4.9‰ and +47‰, respectively).

**FIG. 2.**
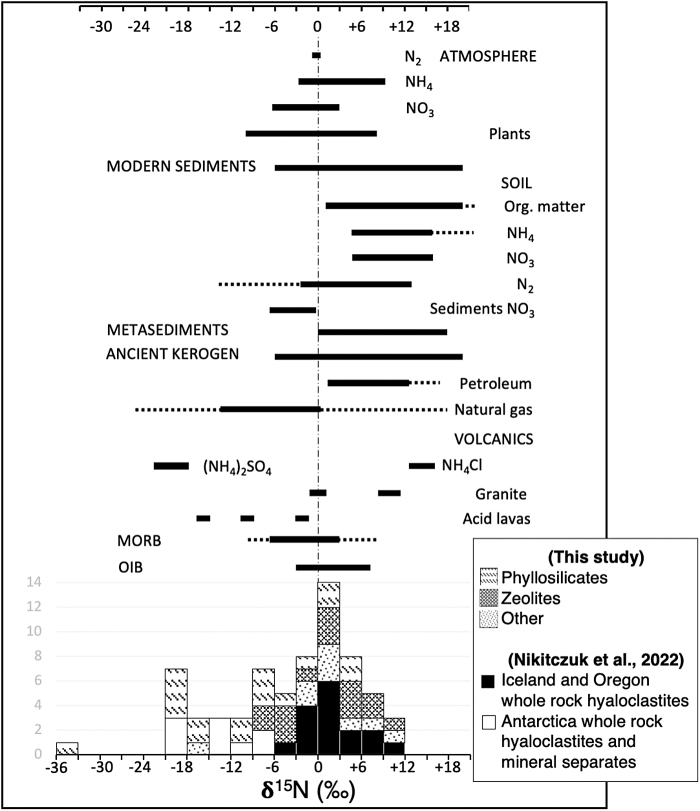
Histogram of δ^15^N values for Mars-analog minerals (phyllosilicates, zeolites, sulfates, silica, feldspathoid, ash, palagonite) and altered Iceland, Oregon, and Antarctica hyaloclastites (from Nikitczuk *et al.*, 2022a) compared with ranges in natural terrestrial materials. Included with “other” are the sulfates alunite, thenardite, and jarosite, amorphous silica, leucite (feldspathoid), palagonite, and volcanic ash. Note that the bulk of the data overlap with the ranges of sedimentary and soil components. Ranges after (Létolle, 1980) and (Cartigny and Marty, 2013). Data not displayed in this plot include fluorapophyllite (RC-298; +47.0‰) and scolecite (8f8-22; +65.4‰).

Out of the 44 samples measured, six have near atmospheric isotope values (*i.e.*, δ^15^N_air_ = 0 ± 2‰). Out of 38 samples with measurable quantities of N, 17 have δ^15^N values similar to that of organic matter in sediments (Fig. 2; δ^15^N between 0‰ and 10‰; see Sweeney *et al.*, 1978; Holloway and Dahlgren, 2002), and 26 are within the range of, or are more positive than, typical MORB values (−5 ± 2‰; see Cartigny and Marty, 2013) or OIBs.

**FIG. 3.**
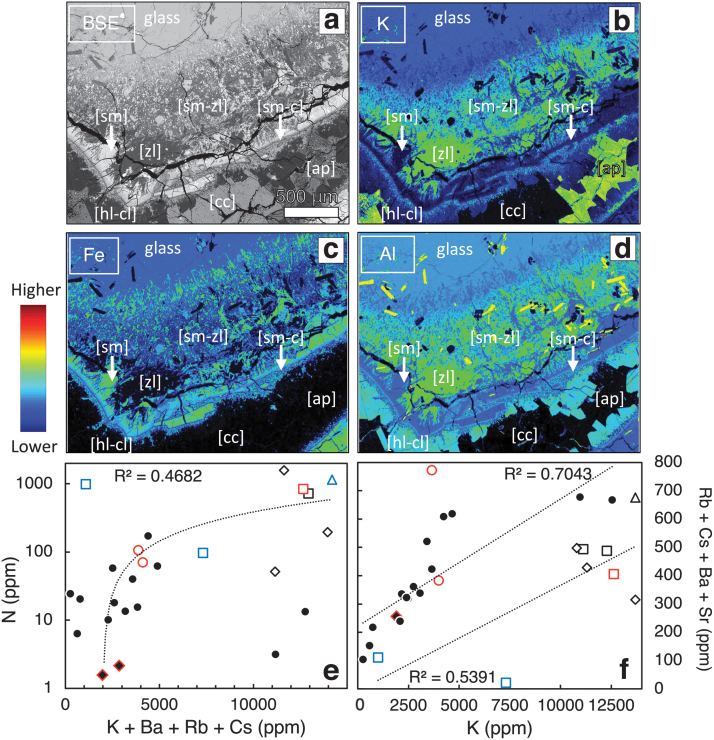
**(A–D)** BSE image and major element maps of altered basaltic andesitic hyaloclastite flow-foot breccia from the Carapace Nunatak, Antarctica (Kirkpatrick Basalt). The BSE image shows a fresh glass core in the top portion of the image that grades irregularly into an alteration zone of smectite- (sm) and zeolite- (zl) replaced glass, a zone of completely smectite-replaced glass nearest to the clast margin, which is also coated in smectites, with the intergranular pore space lined with zeolites (heulandite-clinoptilolite) and infilled with later calcite and apophyllite (phyllosilicate). The high K-Al (and Si) regions (green-turquoise) within the alteration zone and cements represent heulandite-clinoptilolite type zeolites, the high K cement phase at bottom right (green) represents apophyllite, and the higher Fe (and Mg) areas in the alteration zone and along the grain edge (green-light blue) represent nontronite-saponite type smectite clays. The physical separates in Fig. 1 labeled “alteration rinds” correspond to locations such as the smectite-zeolite replaced glass (sm-zl) shown here, whereas separates labeled “zeolite” include locations such as the heulandite-clinoptilolite and apophyllite regions shown here. **(E, F)** Variation diagrams displaying relationships of total K, Ba, Rb, and Cs with N concentrations and of K with total concentrations of alkali and alkaline earth LILEs Rb, Cs, Ba, and Sr, with the latter regarded as fluid-mobile and linked to secondary alteration processes (symbols provided in Fig. 1). Overall positive relationships exist where higher concentrations of alkali LILEs correspond to higher concentrations of N and higher K concentrations are also correlated with higher concentrations of other cations. During alteration processes, cations such as these can become concentrated during the formation of minerals such as phyllosilicates or zeolites. The fixation of N or replacement of cations with N as NH_4_^+^ in crystal lattices of phyllosilicates and zeolites is consistent with the positive correlation with N. Also, significantly more altered Antarctic samples and one Oregon sample with higher proportions of secondary minerals is also consistent with the generally higher concentrations of alkali/alkaline LILEs. Note that the *R*^2^ value from the left variation diagram includes only data for whole rocks, and not mineral separates, whereas the *R*^2^ value for the upper linear regression includes only the data for Iceland whole-rock samples and not the two from Oregon. The y-axis in E is also logarithmic. BSE, back-scattered electron.

Figures 1 and 2 show plots of N concentrations in ppm versus δ^15^N and δ^15^N compared with the ranges of different natural materials, respectively, for the various phyllosilicate, zeolite, sulfate, and amorphous silica-quartz samples obtained in this study and for hyaloclastites from Iceland, Oregon, and Antarctica (data from Nikitczuk *et al.*, 2022a). The major element microprobe data for the hyaloclastites indicate that they contain mixtures of various phyllosilicates that include nontronite-montmorillonite-mica in Antarctic and nontronite-montmorillonite-saponite-beidellite for Iceland samples (Fig. 3; see Nikitczuk *et al.*, 2022a).

Like most of the hyaloclastite samples, all minerals also have higher N concentrations than MORBs or OIBs. The phyllosilicates in this study, especially smectites, have significantly more negative δ^15^N values than all other materials except the Antarctic breccias, with which they overlap in terms of N concentrations and δ^15^N. The zeolites, sulfates, palagonite, and silica varieties, however, overlap with the δ^15^N values of MORBs, OIBs, altered basalts from the literature, and Iceland and Oregon altered basalts (Nikitczuk *et al.*, 2022a), but range to notably more positive values than most of the phyllosilicates analyzed in this study. In addition, many of the phyllosilicates and all zeolites, sulfates, palagonite, and silica-quartz also overlap with the isotope compositions of modern and ancient sediments and soil organic matter (Fig. 2) and are similar to that of the altered basalts, including those from Iceland and Oregon (Nikitczuk *et al.*, 2022a).

### 3.2. Mapping of major element, nitrogen, and carbon concentrations in altered volcanic glasses

Figure 3 shows major element electron microprobe maps of a clast rim and cement in an Antarctic hyaloclastite. One observes a >0.5 mm-wide glass alteration zone and coarse-grained silicate-carbonate cements (see the detailed study by Nikitczuk *et al.*, 2022a). Figure 3 also shows the behavior of N concentration versus the concentrations of different alkali and alkaline-earth elements. It shows co-enrichments of N, and these elements possibly indicating that N occurs largely as NH_4_^+^ in LILE-rich phases such as clay minerals and zeolites (see Kolesov and Geiger, 2006; Geiger and Dachs, 2009; Geiger *et al.*, 2010).

**FIG. 4.**
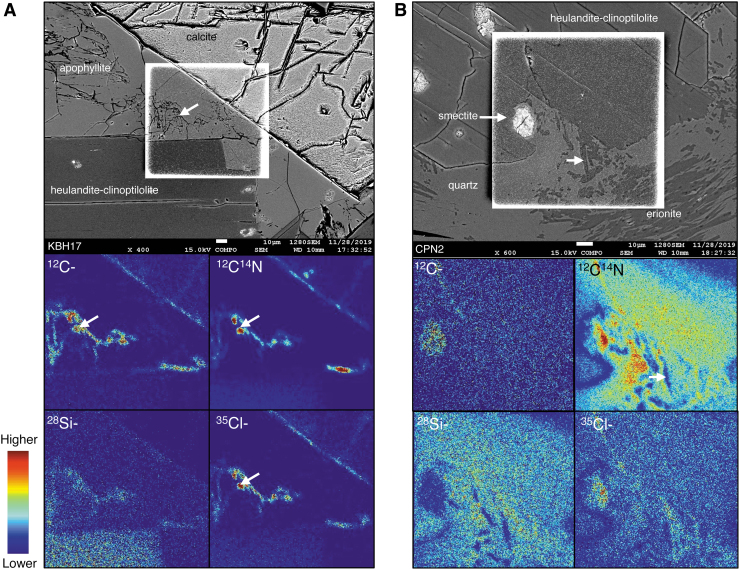
BSE images and ^12^C-, ^12^C^14^N, ^28^Si-, and ^35^Cl- isotope maps from round polished 2.5 cm diameter petrographic thin sections of Antarctic hyaloclastites studied in more detail by Nikitczuk *et al.* (2022a). The BSE images of intergranular cements of Antarctic samples KBH17 **(A)** and CPN2 **(B)** with a post-analysis BSE image of the 80 × 80 μm area over laid in the center. The fractures, grain interfaces, and cleavage traversing the minerals show high contents of ^12^C, ^14^N (detected as ^12^C-^14^N), and ^35^Cl (*e.g.*, white arrows in maps of KBH17). The correlation of C and N with Cl indicates a signal likely produced from epoxy resin used in the thin section. The low signal across the center and at top right of the map indicates that apophyllite and calcite in this sample are likely not storing significant quantities of N. In CPN2, there is an obviously high-^14^N signal in the heulandite-clinoptilolite crystal contrasting with the low signal detected in needle-like erionite crystals.

Element mapping using SIMS in altered hyaloclastites reveals large variations in the N and C concentrations between different authigenic minerals that occur as cements, glass coatings, and glass alteration. Figure 4 shows SIMS N and C element maps obtained for selected areas that contain major secondary phases in rock thin sections of Antarctic hyaloclastites from the Kirkpatrick Basalt. These locations are representative of areas similar to those depicted in the intergranular cemented lower portion in Fig. 3.

The color scales depicted in the SIMS maps represent total ion yield intensity concentrations with warmer and cooler colors plotted using a jet-color scale that corresponds to higher and lower concentrations, respectively. The highest and lowest concentrations in each image are scaled differently, and therefore, the same colors in different maps do not represent the same concentrations of the different elements shown. The K-bearing phases such as heulandite-clinoptilolite, which are expected to contain N as NH_4_^+^, are associated with elevated concentrations of ^12^C^14^N (Fig. 4A, B), with some variability in other zeolites and glass replacement phases. Other K-bearing phases such as apophyllite or erionite, for example, have very low ^12^C^14^N concentrations (Fig. 4A, B). Generally, the highest ^12^C^14^N signals are observed in heulandite-clinoptilolite and clay replacing glass (saponite-nontronite). Zeolites and apophyllite do not contain appreciable ^12^C- or ^13^C-, whereas smectite does show elevated concentrations.

Iceland basaltic tuff samples (Supplementary Fig. S1A, B) show variable ^12^C^14^N concentrations in some palagonites, infilling clays in dissolution channels, pits, vesicles, and zeolite cements. Where multiple layers of clay coatings/cement are visible, for example in vesicles, a difference in N is observed among these layers (*e.g.*, Supplementary Fig. S1B). In several locations within fresh glass of the Iceland basaltic tuff, high ^12^C^14^N signals are detected, but they are accompanied by large variations across different materials in the map area and are coincident with variable ^35^Cl- signals.

Topographic lows within thin sections, such as those from fractures, grain boundaries (*e.g.*, Fig. 4 and Supplementary Fig. S1A), or glass pits, commonly show co-enrichments in C, N, and Cl. It is likely that these elevated and coincident C, N, and Cl concentrations are indicative of the presence of resin; these elements are components of the epoxy resin used to embed the sample. In all instances of N enrichments, however, including those observed in secondary minerals, whether co-enriched in C and/or Cl or not, N consistently shows the highest concentrations. Smectite coatings on glass surfaces also consistently show N enrichments that are commonly coincident with Cl but not with C.

### 3.3. Nitrogen concentrations and isotopic compositions in siliceous deposits

Three samples of amorphous siliceous materials (samples 81233, ICE003, and MAF700) yielded similar N concentrations and δ^15^N of 48–62 ppm and −1.4‰ to +2.1‰, respectively (Table 1; Fig. 1). The δ^15^N values are near Earth atmospheric values (by definition, 0‰), perhaps indicating direct uptake or occlusion (*e.g.*, in fluid inclusions) with only modest amounts of isotopic fractionation. A fourth sample from Mount St. Helens that consists of cristobalite, but also glass and plagioclase, has a far lower N concentration of 0.7 ppm and much lower δ^15^N of −15.4‰.

## 4. Discussion

Most of the K-bearing and micro and meso-porous Mars-surface-analog minerals (Table 1) contain measurable quantities of N, and they show a wide range in both N concentration and δ^15^N (Fig. 1). The isotopic compositions of the various mineral phases analyzed in this study likely reflect the δ^15^N of the fluids present at the time of crystallization or formation, with varying degrees of bioprocessing of this N, with superimposed temperature-dependent fluid-mineral fractionation. Isotopic fractionation in such settings would be dictated by both temperature and the speciation of N in the environments in which the solid phases were crystalized (*e.g.*, as NO_x_, or NH_3_), and as demonstrated by Busigny and Bebout (2013; in particular, see the fractionations among NH_4_^+^, NH_3_, and N_2_), these fractionations can be large at lower temperatures (*i.e.*, below 100°C). The measurements of whole rock altered hyaloclastites by Nikitczuk *et al.*, (2022a) appear to indicate significant secondary N enrichment beyond levels attributable to magmatic/igneous processes.

The N enrichments in minerals studied here, and in the hyaloclastites, at least in part originate from sedimentary/organic-derived components from late low-temperature to hydrothermal or weathering/diagenetic environments. A similar conclusion was reached in a study of altered volcanic glasses of both modern and Mesozoic seafloor (see Bebout *et al.*, 2018) and for altered whole rocks in cored sections of modern seafloor (*e.g.*, Busigny *et al.*, 2005; Li *et al.*, 2007). In aggregate, the minerals analyzed in this study (Table 1) represent the typical products of surface alteration of basalt on Earth, a planet teeming with life. Thus, the results of this study can provide a framework for sampling on the martian surface to maximize the potential of identifying N biosignatures.

### 4.1. Comparison of nitrogen enrichment in Mars-analog minerals with nitrogen residency in altered hyaloclastite whole rocks

Recently, studies focusing on N contents and isotope compositions of silicate rocks have been aimed at understanding mantle and crustal cycling on Earth and possibly other terrestrial planets (see Bebout *et al.*, 2016). They have shown that abiotic and inorganic/organic N can be transferred to secondary phases formed during diagenetic, low-temperature hydrothermal or high pressure-temperature metamorphic fluid-rock interactions that occur during oceanic basalt alteration and subduction processes (Busigny *et al.*, 2005; Li *et al.*, 2007; Halama *et al*., 2010, 2014; Bebout *et al.*, 2013a, 2013b, 2016, 2018; Busigny and Bebout, 2013; Anderson *et al.*, 2019).

Among the samples investigated in this study, significant differences in the range of N concentrations and isotope compositions were observed and notably among different specimens of the same mineral types (Table 1). These include six specimens of apophyllite (0–1754 ppm, δ^15^N = +4.8 to +47.0‰), four of which contained no N, three of halloysite (106–239 ppm, δ^15^N = −19.6‰ to +1.8‰), three kaolinite varieties (17–1086 ppm, δ^15^N = −9.6‰ to +1.8‰), three chabazites (13–85 ppm, δ^15^N = −6.6‰ to 0‰), and three heulandites (0–54 ppm, −2.2‰ to +6.4‰).

These observations of minerals largely formed in volcanic source rock environments highlight the importance of the processes by which, and when, N is being conveyed into these minerals. These differences may also be related to the age of the deposits and, in turn, the overall degree of alteration. In modern palagonitized seafloor basalts and variably altered glasses from Mesozoic ophiolites, Bebout *et al.* (2018), for example, found that altered samples contain higher N concentrations and that the range of concentrations in older samples also extends to higher values (up to 18 ppm N in modern basalts and 53 ppm in Mesozoic ophiolites).

Similarly, Nikitczuk *et al.* (2022a) showed that the N concentrations of whole rock basalts from Iceland (Pleistocene) and Oregon (Pliocene-Pleistocene), along with Antarctic basaltic andesite whole rock and secondary mineral separates (Jurassic; Fig. 1), generally correlate with maturity of alteration stage or reaction progress, observed as greater degrees of crystallinity (Jakobsson and Moore, 1986; Stroncik and Schmincke, 2001, 2002) and deposit age.

The generally lower concentrations of N in Iceland basalts compared with that of the Antarctic basaltic andesites probably resulted from the lower abundances of authigenic minerals in the former, especially zeolite cements and clays, and the difference in zeolite species present and difference in the source of the N. Given that the Iceland and Antarctic localities are interpreted to have been similarly affected by meteoric-sourced, circum-neutral to alkaline waters in open system conditions but at differing temperatures (Nikitczuk *et al.*, 2022a), alteration timing, deposit age, and temperature may be important factors influencing N concentrations and δ^15^N values. Such differences in the formation conditions of the minerals analyzed in this study, among other factors such as water/rock ratio, redox states, and gross quantity of N in the systems, may explain the wide range in N concentrations and δ^15^N values.

Here, it is important to note that, as for the array of minerals from the Earth analyzed in this study, variably altered glasses returned from the martian surface could show a wide range of ages and could similarly have experienced a wide range of environmental conditions during their alteration. What these materials have in common is their derivation largely via alteration of basalt or in other aqueous settings, both known to have been in abundance on the ancient Mars surface. It is likely that the alteration of martian glasses in hydrous conditions would result in a textural and mineralogical progression with age similar to that suggested by Stroncik and Schmincke (2001). Nitrogen uptake and expected residency would be expected to evolve during this progression.

### 4.2. Modes of nitrogen incorporation and storage in various minerals and selected other materials believed to reside on the Mars surface

Major element chemistry of the various phases analyzed in this study has not yet been obtained, and thus the forms of N that are present have not been unequivocally determined. We can, however, use the linkage between the environments in which these phases commonly form (basalt alteration), the whole-rock and *in situ* data obtained for altered hyaloclastites containing the same phases (Nikitczuk *et al.*, 2022a), and other accounts of silicate N to infer the most probable manners in which N has been incorporated and stored in these phases.

In terrestrial igneous and sedimentary crustal rocks, N is typically fixed within secondary and primary K-bearing minerals as NH_4_^+^ (Busigny and Bebout, 2013). Several feldspars (Honma and Itihara, 1981; Krohn *et al.*, 1993; Svensen *et al.*, 2008) and phyllosilicates such as illite-smectite or micas (Williams and Ferrell, 1991; Boyd, 1997; Bobos and Williams, 2017; Jo *et al.*, 2018) from sedimentary environments, for example, have been found to be naturally ammoniated. If N is mainly present as fixed NH_4_^+^, then a good correlation between N and geochemically similar cations such as K^+^ might be expected (Busigny and Bebout, 2013). The lack of visible plagioclase alteration in Iceland and Antarctic hyaloclastites, or any observable relationship with Na or Ca concentrations, suggests that secondary N species do not replace them in the plagioclase structure. The general relationship observed between N concentrations and those of LILE cations (*i.e.*, K, Rb, Cs, Ba, B and U) supports this conclusion (Nikitczuk *et al.*, 2022a).

Iron and Mg concentrated within smectites (*e.g.*, nontronite, saponite in this study, glass coatings and cements in hyaloclastites; Fig. 3) are not replaced by NH_4_^+^, but smectites are known to house elements that are geochemically similar to K mainly as interlayer cations in addition to potential organic molecules. However, deviations from the LILE-N relationship in hyaloclastites and the significantly wide range in N contents and isotope compositions exhibited by the phases in this study could reflect the residency of N in other forms in mineral structures (*e.g.*, N_2_ in channels and cages in microporous mineral phases; Nikitczuk *et al.* 2022a).

Many phases are typically both potassic (Table 1) and/or micro or mesoporous, especially zeolites. Nitrogen can also be supplied as various species (*i.e.*, N_2,_ NH_3_/NH_4_^+^, NO_2_, NO_3_) and be redistributed from different sources under various conditions such as in fluids of alteration processes through degassing of uprising magmas, meteoric-sourced surface waters, or leaching from crustal weathering or sediments. Nitrogen incorporation in addition to fixation in lattices may, thus, occur by occlusion/trapping within structural cavities during formation or by direct adsorption onto charged surfaces or exchange with cations after formation.

Porous zeolite structures and interlamellar smectite clay surfaces have significant ion-exchange/adsorption capacities, whereas zeolites also have molecular sieve properties with cation (*e.g.*, Cs^+^, NH_4_^+^ and Sr^2+^) selectivity. Also, smectites can swell/expand upon hydration and influence interlayer spacing. Volatile gaseous and solution species (*e.g.*, NH_4_^+^, N_2_) within the surrounding environments may, therefore, be readily exchanged with sorbed charged species. The adsorption of N_2_ or exchange of NH_4_^+^ and other cations from solution has been well documented in clinoptilolite, for example (Rožić *et al.*, 2000; Liu *et al.*, 2018; Kennedy *et al.* 2019) and various hydrated clays (*e.g.*, Cornell, 1993; Jurček, 1999; Rožić *et al.*, 2000).

The “confinement effect” of zeolites, that is, the microporous nature of zeolite crystal structures that allows entrapping of molecules within their channels that can subsequently diffuse or participate in physio-adsorption or catalysis (see Derouane, 1987; Derouane *et al.*, 1988; Kolesov and Geiger, 2006; Geiger and Dachs, 2009; Sastre and Corma, 2009) may be important for the incorporation of N during diagenetic processes such as alteration of basalts. Chabazite, for example, is known to occlude several elemental/molecular species in crystal structural voids (*e.g.*, Di Iorio *et al.*, 2020), and N-bearing organic molecules such as small proteins or amino acids may be sequestered (Zimmerman *et al.*, 2004).

The surface area and reactivity of clays also allow for organic compounds within pore fluids (*e.g.*, Kennedy *et al.*, 2002), bacterial cells (Playter *et al.*, 2017), or unsaturated lipids and pigments that are easily oxidized (*e.g.*, Hedges and Keil, 1995) to be preserved through adsorption and encapsulation during deposition. In addition, other microporous minerals, such as melanophlogite (a silica clathrasil) and beryl/cordierite (cyclosilicates), have shown that N_2_ (and possibly NH_4_^+^) in addition to other guest molecules, such as H_2_O, CO_2_, CH_4,_ hydrocarbons, and some organic matter, can be enclathrated or occluded within the structural cavities of their crystal lattices (Bebout *et al.*, 2016; Kortus *et al.*, 2000; Lazzeri *et al.*, 2017; see sketches of zeolite and phyllosilicate atomic structures as insets in Fig. 1) during low-temperature hydrothermal or metamorphic processes.

### 4.3. Nitrogen on early Mars and the potential for atmospheric volatile exchange with martian crustal materials

Various observations of the martian surface suggest that the planet likely had a more substantial atmosphere possibly dominated by CO_2_-N_2_-H_2_O (McKay and Stoker, 1989; Summers and Khare, 2007 and references therein). Noachian pN_2_ may have played an important role in maintaining elevated temperatures and a more reduced, slightly acidic to alkaline, atmosphere containing H_2_O, CO, CH_4_, and NH_3_/NH_4_^+^ (Miller, 2001; Chevrier *et al.*, 2007; Mahaffy *et al.*, 2013; von Paris *et al.*, 2013; Wong *et al.*, 2013; Melwani Daswani *et al.*, 2016; Peretyazhko *et al.*, 2016; Kurokawa *et al.*, 2018; Kajitani *et al.*, 2019; Koike *et al.*, 2020). Although atmospheric NH_3_ is photolytically converted to N_2_ by the absorption of UV radiation, organic aerosols produced by CH_4_ photolysis (Sagan and Chyba, 1997) and other constituents such as H_2_O or CO that absorb similar wavelength UV rays (Hudson, 1974) could have shielded NH_3_ and significantly increased atmospheric residence time. In addition, based on Eu anomalies in shergottite pyroxenes, the martian mantle is highly reducing (Wadhwa, 2001); therefore, N in the mantle is likely to be present as NH_3_ (Libourel *et al.*, 2003).

The global distribution of hydrated mineral deposits on Mars' surface generally consists of two types. Widespread phyllosilicates are mainly observed in ancient Noachian terrains, which indicates their formation very early in Mars' history during the pervasive low-temperature hydrothermal water-igneous rock interaction in alkaline pH subsurface hydrologic systems (Poulet *et al.*, 2005; Bibring *et al.*, 2006; Mustard *et al.*, 2008; Ehlmann *et al.*, 2011; Carter *et al.*, 2013; Sun and Milliken, 2015). A theoretical study by Chevrier *et al.* (2007) is noteworthy as an attempt to calculate surface alteration mineralogy as a function of varying ancient Mars surface conditions. Those authors were focused on the range of conditions affording stabilization of various phyllosilicates, in particular minerals in the smectite group that are typical products of basalt weathering on Earth and apparently on Mars (nontronite and saponite; see Table 1 for the N data for these phases obtained in our study). These thermodynamic calculations indicate that phyllosilicates such as these likely precipitated at alkaline to weakly acidic pH (values of 4–10) perhaps representative of the Noachian but contrasting with the strongly acidic pH that led to the later formation of the extensive sulfate deposits on the martian surface (*e.g.*, jarosite; see the N concentrations and isotopic compositions for various sulfate phases in Table 1).

The theoretical study of Chevrier *et al.* (2007) also indicates that dissolved silica activity strongly influences smectite precipitation, particularly at a lower pH to values as low as 3. Conversely, extensive deposits of sulfates both spatially and temporally represent a climatic change that occurred from the wetter alkaline Noachian to drier acidic environments in the Hesperian (Bibring *et al.*, 2006; Rampe *et al.*, 2020). Some observations, however, indicate that potentially habitable wet and alkaline (*i.e.*, clay forming) conditions or hydrothermal circulation may have existed, at least sporadically, later in Mars' history (*e.g.*, Carter *et al.*, 2013; Grotzinger *et al.*, 2014; Vaniman *et al.*, 2014; Sun and Milliken, 2015). Moreover, measurements by the Curiosity rover are consistent with present-day water exchange between the atmosphere and soil salts, which suggests a widespread existence of liquid water brines (Javier Martín-Torres *et al.*, 2015) beyond equatorial regions. Therefore, phyllosilicates (and zeolites) may have been forming for much of Mars' history and potentially contain a trove of planetary chemical information.

If Earth-like biology ever developed on Mars, microbial fixation processes may have occurred. Experiments subjecting extant terrestrial N-fixing microbes to partial pressures ranging from Earth-like to lower than the current pN_2_ on Mars (0.2 mb) have shown that dinitrogen can be biologically fixed at 5–780 mb. Such pressures could potentially be lower than on primordial Mars (Klingler *et al.*, 1989).

Also, abiotic pathways for N fixation/reduction include impact shock heating, hydrothermal processes, lightning, or cosmic and UV rays. These can dissociate molecular N_2_ to produce N_x_O_x_ species that can be reduced (Mancinelli and McKay, 1988; Summers and Khare, 2007) during catalyzation by FeS (Summers *et al.*, 2012) or other native metals to form NH_4_^+^. Chemical weathering products of Mars surface materials may be directly linked to the physical and chemical incorporation of volatiles such as H_2_O, CO_2_ (Huguenin, 1976), or N_2_/NH_3_/NH_4_^+^ into authigenic minerals. The basalt alteration products palagonite, smectites, and zeolites, which are apparently widespread on Mars' surface (Michalski *et al.*, 2005; Chevrier *et al.*, 2007; Horgan and Bell, 2012), have high specific surface areas and adsorptive capacities (Fanale and Cannon, 1971, 1974, 1978; Fanale *et al.*, 1982) that increase with increasing pressures and temperatures (Fanale and Cannon, 1978; Fanale *et al.*, 1982; Zent *et al.*, 1987).

The Mars regolith possibly played an important role as a strongly adsorbing buffer and exchanger for NH_3_ (Fanale *et al.*, 1982). For example, adsorption and deliquescence (Zent *et al.*, 2010; Harri *et al.*, 2014; Nikolakakos and Whiteway, 2018) and modeling experiments (Mousis *et al.*, 2016) and thermodynamic and powder X-ray diffraction data reveal that, at water vapor pressures and temperatures experienced on the modern Mars' surface, zeolites such as chabazite or clinoptilolite and smectites such as nontronite and montmorillonite can uptake CO_2_ and H_2_O and exist in hydrated states. Such minerals could retain volatiles in micropores (Jänchen *et al.*, 2006) and potentially be as important sinks in the martian subsurface (Mousis *et al.*, 2016) as hidden clathrate reservoirs. Under a more substantial atmosphere that would have allowed more prolonged existence of liquid water, such processes in addition to chemical incorporation by mineral fixation may have been occurring.

### 4.4. The potential of nitrogen in alteration phases on the Mars surface to provide a record of modern and ancient (bio)geochemical processing

Loss of isotopically fractionated N over time may be responsible for Mars' low atmospheric N_2_ concentration and extremely positive δ^15^N value. Analyzing the δ^15^N of ancient materials such as smectite clays or zeolites could help validate whether ancient Mars' atmosphere contained more N with a significantly different isotope composition. In shock-melted glass, for example, from the martian shergottite meteorite ALH84001 with a formation age of 3.9–4.56 Ga (Jagoutz *et al.*, 1994; Turner *et al.*, 1997), comparatively light N (δ^15^N = −30‰ and +4‰) relative to the modern Mars atmosphere has been reported, which supports the N-loss hypothesis.

Consider that the whole N dataset of Mars surface/subsurface-analog minerals (this study) and altered hyaloclastites (Nikitczuk *et al.*, 2022a) are from a terrestrial planet with a well-developed biosphere. However, the isotope compositions straddle Earth's atmosphere (δ^15^N_air_ = 0 ± 2‰) with only a few exceptions (Fig. 2). This range is well within the current uncertainty of the Mars atmosphere (±82‰; Wong *et al.*, 2013), and thus analysis of such materials returned from Mars could provide a relatively accurate approximation of the δ^15^N of the martian atmosphere at the time of their formation. If samples of various ages are obtained, a time series could potentially reveal the timing of the atmospheric loss (shift in δ^15^N), if it, indeed, occurred.

Ammonium in altered basalts may preserve organic matter isotope values that record biogeochemical processes or abiotic igneous processes. Determining the source of N and understanding the processes involved in producing the isotopic composition included within geological materials on Earth requires comparisons with the range of existing reservoirs (Fig. 2). Like many of the minerals analyzed here, several studies of the modern oceanic crust have revealed significant N enrichments complemented by positive shifts in δ^15^N with respect to fresh “mantle-like” values.

Nitrogen enrichments in oceanic crust have been attributed to siting as NH_4_^+^ in potassic secondary phases. Positive δ^15^N values on Earth can reflect fractionations up to 10–30‰ in organic-bearing sediments (*e.g.*, Fig. 2) attributed to bacterially mediated nitrification/denitrification processes or anaerobic ammonium oxidation (Fogel, 2010; Lam and Kuypers, 2011). Therefore, positive δ^15^N shifts and overlapping isotopic compositions of altered rocks and sediments (*e.g.*, Fig. 2) are recognized as resulting from alteration fluids that previously exchanged N with nearby sediments adding sedimentary-organic N to basalts during alteration (Busigny *et al.*, 2005; Li *et al.*, 2007; Bebout *et al.*, 2018). This may be the case for many of the zeolites, phyllosilicates, and other Mars analog materials analyzed here and by Nikitczuk *et al.* (2022a) with more positive δ^15^N and N enrichments relative to MORB and OIB and overlapping δ^15^N values with sediments. Likewise, the altered Late Archaean seafloor (Abitibi greenstone belt) shows similar N incorporation and positive δ^15^N shifts but preserved on time scales of billions of years (Anderson *et al.*, 2019).

Acquiring preserved atmospheric and/or (bio)organic isotopic information requires selecting ancient targets that are as “fresh” as possible from locations with the highest erosion/exposure rates (youngest exposure ages) and that were shielded from highly ionizing galactic and solar cosmic radiation (see Pavlov *et al.*, 2012; Cannon and Mustard, 2015a, 2015b). This is because (1) ancient complex organic molecules are destroyed by cosmic rays in the subsurface (Pavlov *et al.*, 2012; Hays *et al.*, 2017) and affect preservation, and (2) long-term exposure to cosmic rays could significantly alter ^15^N/^14^N or ^13^C/^12^C ratios through spallation nuclear reactions effectively changing the δ^15^N or δ^13^C values (Pavlov *et al.*, 2014).

It appears, though, that the absolute δ^15^N or δ^13^C values are inversely related to the total N or C abundances (Pavlov *et al.*, 2014), and thus if materials with 10s to 1000s of ppm N were obtained, such as the phyllosilicates, zeolites, sulfates, or silica-quartz phases analyzed in this study (Table 1), the isotope shifts would be of notably lower magnitudes than for lower-N materials. It has also been suggested that, on Mars, a geologic rather than an atmospheric N reservoir may be found in ancient N-bearing phyllosilicates that could potentially serve as an abiologic standard against which to identify isotope fractionation patterns (van Zuilen, 2008). On Earth, fractionations are measured as deviations relative to the atmospheric standard (δ^15^N_air_ = 0‰ by definition). Therefore, obtaining ancient, cosmic-ray-shielded, recently exposed, N-bearing minerals may provide such a Mars standard. Such data are even more useful when combined with other isotope systems such as C, with the latter also closely linked to terrestrial biogeochemical cycling (see Banerjee *et al.*, 2006).

## 5. Conclusions

As planetary exploration and Mars sample-return advance, it is crucial that scientifically rewarding targets are selected that are worthy of the meticulous and expensive efforts such missions employ. By investigating the concentrations and isotope compositions of N, an element of biogeochemical significance, in mainly low-temperature basaltic alteration minerals and other phases that are recognized as existent or potentially present on Mars' surface and comparing them with that of well-characterized altered hyaloclastites, we have established that most of these potassic and/or micro and mesoporous materials are natural receptacles for N once in the atmosphere.

Given that some phases on Mars such as certain phyllosilicates (*i.e.*, nontronite) may have formed deep in Mars' past and, along with other phases, continued to form at various geologic points in time, nitrogenous materials may provide a unique potential in preserving planetary N records that are valuable to sample-return science goals. Variably altered basaltic glasses are enriched in N relative to MORB or OIB but show a wide range of concentrations and isotopic compositions, many of which overlap δ^15^N values of sedimentary/organic components. This enrichment is indicative of processes beyond magmatic degassing that includes supply during low-temperature to hydrothermal alteration. Silicate N is commonly fixed as NH_4_^+^ replacing K^+^, as indicated by relationships between N and alkali/LILE cations (*i.e.*, K, Rb, Cs, Ba, Fig. 3) in altered Iceland, Oregon, and Antarctic hyaloclastites. However, various minerals and phases in this study that do not typically contain K in their crystal structures still contain N and show differences between different specimens of the same mineral types. This, in combination with the scatter in cation correlations and the range in N concentrations and δ^15^N, suggests the existence of several potential retention sites and retained N species, such as exchanged or occluded/trapped or encapsulated NH_3_/NH_4_^+^ (including organic molecules), or N_2_ within structural cavities or interlayer surfaces.

Although only nitrates have been found in Mars' surface materials to date, given that terrestrial basalt alteration phases and other minerals can be significant N repositories and that Noachian Mars may have contained more atmospheric N_2_, H_2_O, or possibly NH_3_, analogous speciation and siting in similar hydrous mineral phases at or below the Mars' surface in forms other than nitrates may have occurred. We propose that evidence of the existence of N-fixing biological processes, if they developed on Mars, or information regarding the abiotic chemical evolutionary past of Mars, could exist as mineralogically preserved organic or fixed N and its stable isotope composition in phyllosilicates, zeolites, siliceous materials, hydrated sulfates, or nitrate-bearing deposits.

Determining the inventory, form, distribution, and storage phases of N is a critical factor for understanding the probability of life originating and evolving on another planetary body such as Mars. Future Mars' surface lander or rover missions should consider N inclusion in phases associated with altered basalts to aid in guiding appropriate sample selection, especially for return to Earth. The N concentrations and isotopic compositions in minerals should, however, be considered in context with other geological features (sedimentary deposits, igneous intrusions/lava flows, relationships to paleo-environments such as lakes).

Future contributions to this work should consider (1) major and trace element compositions of the minerals, (2) analysis of local sedimentary materials and environmental parameters such as water chemistry and temperature contributing to the alteration of specific glasses/basalts, (3) delineating the specific forms of N present including organic molecules, and (4) combining other isotope systems with N to evaluate magmatic and organic source contributions (*e.g.*, ^40^Ar/^36^Ar, ^4^He/^40^Ar, ^13^C/^12^C) or water chemistry and temperature effects (*e.g.*, H/D and O).

## Supplementary Material

Supplemental data

## Abbreviations Used

BSE: back-scattered electron
c: carbonate
CAS: College of Arts and Sciences
CSA: Canadian Space Agency
EPMA: electron probe microanalyzer
f: feldspar
MORB: mid-ocean ridge basalts
MSL: Mars Science Laboratory
N: nitrogen
N_2_: molecular nitrogen
OIB: ocean-island basalts
OISS: Office of International Students and Scholars
p: phyllosilicate
si: silica
SIMS: secondary ion mass spectrometry
STP: standard temperature and pressure
su: sulfate
z: zeolite

## Notes

Associate Editor: Nita Sahai

## References

[B1] Anderson LD, Bebout GE, Izawa MRM, *et al.* (2019) Chemical alteration and preservation of sedimentary/organic nitrogen isotope signatures in a 2.7 Ga seafloor volcanic sequence. Int J Astrobiol 18, 235–250.

[B2] Arvidson RE, Poulet F, Bibring JP, *et al.* (2005) Spectral reflectance and morphologic correlations in eastern Terra Meridiani, Mars. Science 307, 1591–1594. 15718425 10.1126/science.1109509

[B3] Arvidson RE, Ruff SW, Morris RV, *et al.* (2008) Spirit Mars Rover Mission to the Columbia Hills, Gusev Crater: mission overview and selected results from the Cumberland Ridge to Home Plate. J Geophys Res E Planets 113, E12S33.

[B4] Bandfield JL, Hamilton VE, and Christensen PR. (2000) A global view of martian surface compositions from MGS-TES. Science 287, 1626–1630.

[B5] Banerjee NR, Furnes H, Muehlenbachs K, *et al.* (2006) Preservation of ∼3.4-3.5 Ga microbial biomarkers in pillow lavas and hyaloclastites from the Barberton Greenstone Belt, South Africa. Earth Planet Sci Lett 241, 707–722.

[B6] Bebout GE, Idleman BD, Li L, *et al.* (2007) Isotope-ratio-monitoring gas chromatography methods for high-precision isotopic analysis of nanomole quantities of silicate nitrogen. Chem Geol 240, 1–10.

[B7] Bebout GE, Banerjee NR, Izawa MRM, *et al.* (2018) Nitrogen concentrations and isotopic compositions of seafloor-altered terrestrial basaltic glass: implications for astrobiology. Astrobiology 18, 330–342. 29106312 10.1089/ast.2017.1708 PMC5867513

[B8] Bebout GE, Fogel ML, and Cartigny P (2013a) Nitrogen and its (biogeocosmo) chemical cycling. Elements 9, 333–374.

[B9] Bebout GE, Fogel ML, and Cartigny P (2013b) Nitrogen: highly volatile yet surprisingly compatible. Elements 9, 333–338.

[B10] Bebout GE, Lazzeri KE, and Geiger CA (2016) Pathways for nitrogen cycling in Earth's crust and upper mantle: a review and new results for microporous beryl and cordierite. Am Mineral DOI: 10.2138/am-2016-5363.

[B11] Bibring JP, Langevin Y, Mustard JF, *et al.* (2006) Global mineralogical and aqueous Mars history derived from OMEGA/Mars express data. Science 312, 400–404. 16627738 10.1126/science.1122659

[B12] Bishop JL, Banin A, Mancinelli RL, *et al.* (2002) Detection of soluble and fixed NH_4_^+^ in clay minerals by DTA and IR reflectance spectroscopy: a potential tool for planetary surface exploration. Planet Space Sci 50, 11–19.

[B13] Bobos I and Williams LB (2017) Boron, lithium and nitrogen isotope geochemistry of NH_4_^-^illite clays in the fossil hydrothermal system of Harghita Bãi, East Carpathians, Romania. Chem Geol 473, 22–39.

[B14] Boyd SR (1997) Determination of the ammonium content of potassic rocks and minerals by capacitance manometry: a prelude to the calibration of FTIR microscopes. Chem Geol 137, 57–66.

[B15] Boyd SR (2001) Nitrogen in future biosphere studies. Chem Geol 176, 1–30.

[B16] Brinkmann RT (1971) Mars: has N escaped? *Science* 174, 944–945. 10.1126/science.174.4012.944 17773192

[B17] Bristow TF, Rampe EB, Achilles CN, *et al.* (2018) Clay mineral diversity and abundance in sedimentary rocks of Gale crater, Mars. Sci Adv 4, 1–9. 10.1126/sciadv.aar3330 PMC5990309 29881776

[B18] Busigny V and Bebout GE (2013) Nitrogen in the silicate Earth: speciation and isotopic behaviour during mineral-fluid interactions. Elements 9, 353–358.

[B19] Busigny V, Ader M, and Cartigny P (2005) Quantification and isotopic analysis of nitrogen in rocks at the ppm level using sealed tube combustion technique: a prelude to the study of altered oceanic crust. Chem Geol 223, 249–258.

[B20] Canfield DE, Glazer AN, and Falkowski PG (2010) The evolution and future of Earth's nitrogen cycle. Science 330, 192–196. 20929768 10.1126/science.1186120

[B21] Cannon KM and Mustard JF (2015a) Follow the glass: preservation and colonization potential of Martian glass-bearing impactites. Lunar and Planetary Science Conference Abstracts (1900). https://www.hou.usra.edu/meetings/lpsc2015/pdf/1900.pdf (last accessed October 27, 2017).

[B22] Cannon KM and Mustard JF (2015b) Preserved glass-rich impactites on Mars. Geology 43, 635–638.

[B23] Cannon KM, Mustard JF, and Salvatore MR. (2015) Alteration of immature sedimentary rocks on Earth and Mars: recording aqueous and surface-atmosphere processes. Earth Planet Sci Lett 417, 78–86.

[B24] Capone DG, Popa R, Flood B, *et al.* (2006) Follow the nitrogen. Science 312, 708–709. 16675690 10.1126/science.1111863

[B25] Carrozzo FG, Di Achille G, Salese F, *et al.* (2017) Geology and mineralogy of the Auki Crater, Tyrrhena Terra, Mars: a possible post impact-induced hydrothermal system. Icarus 281, 228–239.

[B26] Carter J, Poulet F, Bibring JP, *et al.* (2013) Hydrous minerals on Mars as seen by the CRISM and OMEGA imaging spectrometers: updated global view. J Geophys Res E Planets 118, 831–858.

[B27] Cartigny P and Marty B (2013) Nitrogen isotopes and mantle geodynamics: the emergence of life and the atmosphere-crust-mantle connection: Elements 9, 359–366.

[B28] Cartigny P, Jendrzejewski N, Pineau F, *et al.* (2001) Volatile (C, N, Ar) variability in MORB and the respective roles of mantle source hterogeneity and degassing: the case of the Southwest indian Ridge. Earth Planet Sci Lett 194, 241–257.

[B29] Chevrier V, Poulet F, and Bibring JP (2007) Early geochemical environment of Mars as determined from thermodynamics of phyllosilicates. Nature 448, 60–63. 17611538 10.1038/nature05961

[B30] Cloutis EA, Mann P, Izawa MRM, *et al.* (2015) The Canadian space agency planetary analogue materials suite. Planet Space Sci 119, 155–172.

[B31] Cornell RM (1993) Adsorption of cesium on minerals: a review. J Radioanal Nuclear Chem Articles 171, 483–500.

[B32] Derouane EG (1987) The energetics of sorption by molecular sieves: surface curvature effects. Chem Phys Lett 142, 200–204.

[B33] Derouane EG, Andre JM, and Lucas AA (1988) Surface curvature effects in physisorption and catalysis by microporous solids and molecular sieves. J Catalysis 110, 58–73.

[B34] De Vet SJ, Merrison JP, Mittelmeijer-Hazeleger MC, *et al.* (2014) Effects of rolling on wind-induced detachment thresholds of volcanic glass on Mars. Planet Space Sci 103, 205–218.

[B35] Di Iorio JR, Li S, Jones CB, *et al.* (2020) Cooperative and competitive occlusion of organic and inorganic structure-directing agents within chabazite zeolites influences their aluminum arrangement. J Am Chem Soc 142, 4807–4819. 32053365 10.1021/jacs.9b13817

[B36] Ehlmann BL and Edwards CS (2014) Mineralogy of the martian surface. Ann Rev Earth Planet Sci 42, 291–315.

[B37] Ehlmann BL, Mustard JF, Murchie SL, *et al.* (2008) Orbital identification of carbonate-bearing rocks on Mars. Science 322, 1828–1832. 19095939 10.1126/science.1164759

[B38] Ehlmann BL, Mustard JF, Swayze GA, *et al.* (2009) Identification of hydrated silicate minerals on Mars using MRO-CRISM: geologic context near Nili Fossae and implications for aqueous alteration. J Geophys Res E Planets DOI: 10.1029/2009JE003339.

[B39] Ehlmann BL, Mustard JF, Murchie SL, *et al.* (2011) Subsurface water and clay mineral formation during the early history of Mars. Nature 479, 53–60. 22051674 10.1038/nature10582

[B40] Exley RA, Boyd SR, Mattey DP. et al. (1986/87) Nitrogen isotope geochemistry of bastic glasses: implications for mantle degassing and structure? *Earth Planet Sci Lett* 81, 163–174.

[B41] Fanale FP and Cannon WA (1971) Adsorption on the martian regolith. Nature 230, 502–504.

[B42] Fanale FP and Cannon WA (1974) Exchange of adsorbed H_2_O and CO_2_ between the regolith and atmosphere of Mars caused by changes in surface insolation. J Geophys Res 79, 3397–3402.

[B43] Fanale FP and Cannon WA (1978) Mars: the role of the regolith in determining atmospheric pressure and the atmosphere's response to insolation changes. J Geophys Res 83(B5), 2321.

[B44] Fanale, Fraser P, Salvail JR, Bruce Banerdt W, *et al.* (1982) Mars: the regolith-atmosphere-cap system and climate change. Icarus 50, 381–407.

[B45] Fogel ML (2010) Variations of abundances of nitrogen isotopes in nature. In: The Encyclopedia of Mass Spectrometry, Volume 5. Elsevier, Amsterdam, pp 842–852.

[B46] Fox JL and Dalgarno A (1983) Nitrogen escape from Mars. J Geophys Res Space 88, 9027–9032.

[B47] Geiger CA and Dachs E (2009) Quasi-ice-like Cp behavior of molecular H2O in hemimorphite Zn_4_Si_2_O_7_(OH)_2_H_2_O: Cp and entropy of confined H_2_O in microporous silicates. Am Mineral 94, 634–637.

[B48] Geiger CA, Dachs E, Dalconi MC, *et al.* (2010) Molecular H_2_O in armenite, BaCa_2_Al_6_Si_9_O_3_0 · 2H_2_O, and epididymite, Na_2_Be_2_Si_6_O_15_·H_2_O: heat capacity, entropy and local-bonding behavior of confined H_2_O in microporous silicates. Geochim Cosmochim Acta 74, 5202–5215.

[B49] Gendrin A, Mangold N, Bibring JP, *et al.* (2005) Sulfates in Martian layered terrains: The OMEGA/Mars express view. Science 307, 1587–1591. 15718429 10.1126/science.1109087

[B50] Gregg TKP and Williams SN (1996) Basaltic volcanism study project. Icarus 122, 397–405.

[B51] Grotzinger JP, Sumner DY, Kah LC, *et al.* (2014) A habitable fluvio-lacustrine environment at Yellowknife Bay, Gale crater, Mars. Science 343, 1–15. 10.1126/science.1242777 24324272

[B52] Halama R, Bebout GE, John T, *et al.* (2010) Nitrogen recycling in subducted oceanic lithosphere: the record in high- and ultrahigh-pressure metabasaltic rocks. Geochim Cosmochim Acta 74, 1636–1652.

[B53] Halama R, Bebout GE, John T, *et al.* (2014) Nitrogen recycling in subducted mantle rocks and implications for the global nitrogen cycle. Int J Earth Sci 103, 2081–2099.

[B54] Halldórsson SA, Hilton DR, Barry PH, *et al.* (2016) Recycling of crustal material by the Iceland mantle plume: new evidence from nitrogen elemental and isotope systematics of subglacial basalts. Geochim Cosmochim Acta 176, 206–226.

[B55] Harri AM, Genzer M, Kemppinen O, *et al.* (2014) Mars Science Laboratory relative humidity observations: initial results. J Geophys Res E Planets 119, 2132–2147. 10.1002/2013JE004514 PMC4508910 26213667

[B56] Hays LE, Graham HV, Des Marais DJ, *et al.* (2017) Biosignature preservation and detection in mars analog environments. Astrobiology 17, 363–400. 28177270 10.1089/ast.2016.1627 PMC5478115

[B57] Hedges JI and Keil RG (1995) Sedimentary organic matter preservation: an assessment and speculative synthesis. Marine Chemi 49, 81–115.

[B58] Holloway JM and Dahlgren RA (2002) Nitrogen in rock: occurrences and biogeochemical implications. Glob Biogeochem Cycles 16, 1118.

[B59] Honma H and Itihara Y (1981) Distribution of ammonium in minerals of metamorphic and granitic rocks. Geochim Cosmochim Acta 45, 983–988.

[B60] Horgan B and Bell JF (2012) Widespread weathered glass on the surface of Mars. Geology 40, 391–394.

[B61] Hudson RD (1974) Absorption cross sections of stratospheric molecules. Canadian J Chem 52, 1465–1478.

[B62] Huguenin RL (1976) Mars: chemical weathering as a massive volatile sink. Icarus 28, 203–212.

[B63] Jagoutz AS, Voggel JD, and Wänke. (1994) ALH84001: alien or progenitor of the SNC family? Meteorit Sco 29, 478–479.

[B64] Jakobsson SP and Moore JG (1986) Hydrothermal minerals and alteration rates at Surtsey volcano, Iceland. Geol Soc Am Bull 97, 648–659.

[B65] Jakosky BM, Pepin RO, Johnson RE, *et al.* (1994) Mars atmospheric loss and isotopic fractionation by solar-wind-induced sputtering and photochemical escape. Icarus 111, 271–288.

[B66] Jänchen J, Bish DL, Möhlmann DTF, *et al.* (2006) Investigation of the water sorption properties of Mars-relevant micro- and mesoporous minerals. Icarus 180, 353–358.

[B67] Jaramillo EA, Royle SH, Claire MW, *et al.* (2019) Indigenous organic-oxidized fluid interactions in the Tissint Mars meteorite. Geophys Res Lett 46, 3090–3098.

[B68] Javier Martín-Torres F, Zorzano MP, Valentín-Serrano P, *et al.* (2015) Transient liquid water and water activity at Gale crater on Mars. Nat Geosci 8, 357–361.

[B69] Jo J, Yamanaka T, Kashimura T, *et al.* (2018) Mineral nitrogen isotope signature in clay minerals formed under high ammonium environment conditions in sediment associated with ammonium-rich sediment-hosted hydrothermal system. Geochem J 52, 1–17.

[B70] Jurček P (1999) Study of sorption and diffusion processes in natural zeolites. Czechoslov J Phys 49, 657–664.

[B71] Kajitani I, Tanabe G, Nakada R, *et al.* (2019) Finding of oxidized sulfur species in carbonates from a Martian meteorite Allan Hills 84001 using μ-XANES. In: Ninth International Conference on Mars, p 6446. 10.22201/fq.18708404e.2004.3.66178

[B72] Kennedy DA, Mujčin M, Abou-Zeid C, *et al.* (2019) Cation exchange modification of clinoptilolite –thermodynamic effects on adsorption separations of carbon dioxide, methane, and nitrogen. Microporous Mesoporous Mater 274, 327–341.

[B73] Kennedy MJ, Pevear DR, and Hill RJ (2002) Mineral surface control of organic carbon in black shale. Science 295, 657–660. 11809966 10.1126/science.1066611

[B74] Klingler JM, Mancinelli RL, and White MR (1989) Biological nitrogen fixation under primordial Martian partial pressures of dinitrogen. Adv Space Res 9, (6)173–176. 11537369 10.1016/0273-1177(89)90225-1

[B75] Koike M, Nakada R, Kajitani I, *et al.* (2020) In-situ preservation of nitrogen-bearing organics in Noachian Martian carbonates. Nat Commun 11, 1–7. 32332762 10.1038/s41467-020-15931-4 PMC7181736

[B76] Kolesov BA and Geiger CA (2006) Behaviour of H_2_O molecules in the channels of natrolite and scolecite: a raman and IR spectroscopic investigation of hydrous microporous silicates. Am Mineral 91, 1039–1048.

[B77] Kortus J, Irmer G, Monecke J, *et al.* (2000) Influence of cage structures on the vibrational modes and Raman activity of methane. Model Simul Mater Sci Eng 8, 403–411.

[B78] Kounaves SP, Carrier BL, O'Neil GD, *et al.* (2014) Evidence of martian perchlorate, chlorate, and nitrate in Mars meteorite EETA79001: implications for oxidants and organics. Icarus 229, 206–213.

[B79] Kraft MD, Michalski JR, and Sharp TG (2003) Effects of pure silica coatings on thermal emission spectra of basaltic rocks: Considerations for Martian surface mineralogy. Geophys Res Lett 30 DOI: 10.1029/2003GL018848.

[B80] Krohn MD, Kendall C, Evans JR, et al. (1993) Relations of ammonium minerals at several hydrothermal systems in the western U.S. *J Volcanol Geotherm Res* 56, 401–413.

[B81] Kurokawa H, Kurosawa K, and Usui, T (2018) A lower limit of atmospheric pressure on early Mars inferred from nitrogen and argon isotopic compositions. Icarus 299, 443–459.

[B82] Lam P and Kuypers MMM (2011) Microbial nitrogen cycling pocesses in oxygen minimum zones. Ann Rev Marine Sci 3, 317–345. 10.1146/annurev-marine-120709-142814 21329208

[B83] Lazzeri KE (2012) Storage of Nitrogen in Silicate Minerals and Glasses. Masters thesis, Lehigh University.

[B84] Lazzeri KE, Bebout GE, and Geiger CA (2017) Nitrogen and carbon concentrations and isotopic compositions of the silica clathrate melanophlogite. Am Mineral 102, 686–689.

[B85] Létolle R (1980) Nitrogen-15 in the natural environment. In: Handbook of Environmental Isotope Geochemistry, Volume 1 A, edited by P. Fritz and J.C. Fontes, Elsevier Scientific Publishing Company, Amsterdam, Netherlands, pp 407–433. 10.1016/b978-0-444-41780-0.50016-x

[B86] Li L, Bebout GE, and Idleman BD (2007) Nitrogen concentration and δ^15^N of altered oceanic crust obtained on ODP Legs 129 and 185: insights into alteration-related nitrogen enrichment and the nitrogen subduction budget. Geochim Cosmochim Acta 71, 2344–2360.

[B87] Libourel G, Marty B, and Humbert F (2003) Nitrogen solubility in basaltic melt. Part I. Effect of oxygen fugacity. Geochim Cosmochim Acta 67, 4123–4135.

[B88] Liu X, Xie W, Cui X, *et al.* (2018) Clinoptilolite tailored to methane or nitrogen selectivity through different temperature treatment. Chem Phys Lett 707, 75–79.

[B89] Mahaffy PR, Webster CR, Atreya SK, *et al.* (2013) Abundance and isotopic composition of gases in the martian atmosphere from the Curiosity Rover. Science 341, 263–266. 23869014 10.1126/science.1237966

[B90] Mancinelli RL (1996) The search for nitrogen compounds on the surface of Mars. Adv Space Res doi: 10.1016/0273-1177(96)00113-5. 12577965

[B91] Mancinelli RL and Banin A (2003) Where is the nitrogen on Mars? *Int J Astrobiol* 2, 217–225.

[B92] Mancinelli RL and McKay CP (1988) The evolution of nitrogen cycling. Orig Life Evol Biosph 18, 311–325. 11538360 10.1007/BF01808213

[B93] Marty B and Dauphas N (2003) The nitrogen record of crust-mantle interaction and mantle convection from Archean to present. Earth Planet Sci Lett 206, 397–410.

[B94] Marty B and Humbert F (1997) Nitrogen and argon isotopes in oceanic basalts. Earth Planet Sci Lett 152, 101–112.

[B95] Marty B and Zimmermann L (1999) Volatiles (He, C, N, Ar) in mid-ocean ridge basalts: assessment of shallow-level fractionation and characterization of source composition. Geochim Cosmochim Acta 63, 3619–3633.

[B96] McElroy MB (1972) Mars: an evolving atmosphere. Science 175, 443–445. 17731370 10.1126/science.175.4020.443

[B97] McElroy MB, Ling Yung Y, Nier AO. (1976) Isotopic composition of nitrogen: implications for the past history of Mars' atmosphere. Science 194:70–72. 17793081 10.1126/science.194.4260.70

[B98] McKay CP and Stoker CR (1989) The early environment and its evolution on Mars: implication for life. Rev Geophys 27, 189–214.

[B99] Melwani Daswani M, Schwenzer SP, Reed MH, *et al.* (2016) Alteration minerals, fluids, and gases on early Mars: predictions from 1-D flow geochemical modeling of mineral assemblages in meteorite ALH 84001. Meteor Planet Sci 51, 2154–2174.

[B100] Michalski JR, Kraft MD, Sharp TG, *et al.* (2005) Mineralogical constraints on the high-silica martian surface component observed by TES. Icarus 174, 161–177.

[B101] Miller IJ (2001) Early martian atmosphere and biogenesis. Chemistry Preprint Archive. Lower Hutt: Elsevier.

[B102] Milliken RE, Swayze GA, Arvidson RE, *et al.* (2008) Opaline silica in young deposits on Mars. Geology 36, 847–850.

[B103] Ming DW and Gooding JL (1988) Zeolites on Mars: possible environmental indicators in soils and sediments. In: Lunar and Planetary Institute Conference Abstracts, pp 124–125.

[B104] Morris RV, Ruff SW, Gellert R, *et al.* (2010) Identification of carbonate-rich outcrops on Mars by the Spirit rover. Science 329, 421–424. 20522738 10.1126/science.1189667

[B105] Mousis O, Simon JM, Bellat JP, *et al.* (2016) Martian zeolites as a source of atmospheric methane. Icarus 278, 1–6.

[B106] Murray HH (2006) Chapter 2: structure and composition of the clay minerals and their physical and chemical properties. In: Developments in Clay Science, Volume 2, edited by H.H. Murray, Elsevier, Amsterdam, pp 7–31.

[B107] Mustard JF, Murchie SL, Pelkey SM, *et al.* (2008) Hydrated silicate minerals on Mars observed by the Mars Reconnaissance Orbiter CRISM instrument. Nature DOI: 10.1038/nature07097. 18633411

[B108] Nikitczuk MP, Bebout GE, Ota T, *et al.* (2022a) Nitrogenous altered volcanic glasses as targets for Mars sample return: examples from Antarctica and Iceland. J Geophys Res Planets 127, e2021JE007052.

[B109] Nikitczuk MP, Bebout GE, Ota T, et al. (2022b) Major and trace element and N concentrations and isotope compositions of altered basaltic and basaltic andesitic hyaloclastite whole-rocks, mineral separates and thin sections from Antarctica, Iceland and Oregon, U.S.A., Version 1.0. Interdisc Earth Data Alliance (IEDA) DOI: 10.26022/IEDA/112220.

[B110] Nikolakakos G and Whiteway JA (2018) Laboratory study of adsorption and deliquescence on the surface of Mars. Icarus 308, 221–229.

[B111] Niles PB, Catling DC, Berger G, *et al.* (2013) Geochemistry of carbonates on Mars: implications for climate history and nature of aqueous environments. Space Sci Rev 174 (1–4), 301–328.

[B112] Papineau D, Mojzsis SJ, Karhu JA, *et al.* (2005) Nitrogen isotopic composition of ammoniated phyllosilicates: case studies from Precambrian metamorphosed sedimentary rocks. Chem Geol 216, 37–58.

[B113] Pavlov AA, Vasilyev G, Ostryakov VM, *et al.* (2012) Degradation of the organic molecules in the shallow subsurface of Mars due to irradiation by cosmic rays. Geophys Res Lett 39, 5–9.

[B114] Pavlov AA, Pavlov AK, Ostryakov VM, *et al.* (2014) Alteration of the carbon and nitrogen isotopic composition in the Martian surface rocks due to cosmic ray exposure. J Geophys Res Planets 119, 1390–1402.

[B115] Peretyazhko TS, Sutter B, Morris RV, *et al.* (2016) Fe/Mg smectite formation under acidic comditions on early Mars. Geochim Cosmochim Acta 173, 37–49. 10.1016/j.gca.2017.10.004 PMC7427815 32801388

[B116] Playter T, Konhauser K, Owttrim G, *et al.* (2017) Microbe-clay interactions as a mechanism for the preservation of organic matter and trace metal biosignatures in black shales. Chem Geol 459, 75–90.

[B117] Poulet F, Bibring JP, Mustard JF, *et al.* (2005) Phyllosilicates on Mars and implications for early martian climate. Nature 438, 623–627. 16319882 10.1038/nature04274

[B118] Poulet F, Gomez C, Bibring JP, *et al.* (2007) Martian surface mineralogy from observatoire pour la minéralogie, l'Eau, les Glaces et l'Activité on board the Mars Express spacecraft (OMEGA/MEx): global mineral maps. J Geophys Res E Planets 112, 1–15.

[B119] Rampe EB, Blake DF, Bristow, *et al.* (2020) Mineralogy and geochemistry of sedimentary rocks and eolian sediments in Gale crater, Mars: a review after six earth years of exploration with curiosity. Geochemistry 80, DOI: 10.1016/j.chemer.2020.125605.

[B120] Rožić M, Cerjan-Stefanović Š, Kurajica S, *et al.* (2000) Removal of ammonical nitrogen by electrocoagulation method. Water Res 34, 3675–3681.

[B121] Ruff SW (2004) Spectral evidence for zeolite in the dust on Mars. Icarus 168, 131–143.

[B122] Ruff SW and Farmer JD (2016) Silica deposits on Mars with features resembling hot spring biosignatures at El Tatio in Chile. Nat Commun 7, 1–10. 10.1038/ncomms13554 PMC5473637 27853166

[B123] Ruff SW, Campbell KA, Van Kranendonk MJ, *et al.* (2020) The case for ancient hot springs in Gusev Crater, Mars. Astrobiology 20, 475–499. 31621375 10.1089/ast.2019.2044 PMC7133449

[B124] Sætre C, Hellevang H, Riu L, *et al.* (2019) Experimental hydrothermal alteration of basaltic glass with relevance to Mars. Meteor Planet Sci 54, 357–378.

[B125] Sagan C and Chyba C (1997) The early faint sun paradox: organic shielding of ultraviolet-labile greenhouse gases. Science 276, 1217–1221. 11536805 10.1126/science.276.5316.1217

[B126] Sakai H, Des Marais DJ, Ueda A, *et al.* (1984) Concentrations and isotope ratios of carbon, nitrogen and sulfur in ocean-floor basalts. Geochim Cosmochim Acta 48, 2433–2441. 11540821 10.1016/0016-7037(84)90295-3

[B127] Sastre G and Corma A (2009) The confinement effect in zeolites. J Mol Catal A Chem 305, 3–7.

[B128] Schmidt ME, Farrand WH, Johnson JR, *et al.* (2009) Spectral, mineralogical, and geochemical variations across Home Plate, Gusev Crater, Mars indicate high and low temperature alteration. Earth Planet Sci Lett 281, 258–266.

[B129] Schmidt ME, Campbell JL, Gellert R, *et al.* (2014) Geochemical diversity in first rocks examined by the curiosity rover in gale crater: evidence for and significance of an alkali and volatile-rich igneous source. J Geophys Res E Planets 119, 64–81.

[B130] Squyres SW, Arvidson RE, Iii JF. B., *et al.* (2004a) The opportunity rover's Athena science investigation at Meridiani Planum, Mars. Science 306, 1698–1703. 15576602 10.1126/science.1106171

[B131] Squyres SW, Grotzinger JP, Arvidson RE, *et al.* (2004b) In situ evidence for an ancient aqueous environment at Meridiani Planum, Mars. Science 306, 1709–1714. 15576604 10.1126/science.1104559

[B132] Squyres SW, Oren A, Clark BC, *et al.* (2007) Pyroclastic activity at home plate in Gusev Crater, Mars. Science 36, 738–742. 10.1126/science.1139045 17478719

[B133] Squyres SW, Arvidson RE, Ruff S, *et al.* (2008) Detection of silica-rich deposits on Mars. Science 320, 1063–1067. 18497295 10.1126/science.1155429

[B134] Steller LH, Nakamura E, Ota T, *et al.* (2019) Boron isotopes in the Puga geothermal system, India, and their implications for the habitat of early life. Astrobiology 19, 1459–1473. 31287717 10.1089/ast.2018.1966

[B135] Stern JC, Sutter B, Freissinet C, *et al.* (2015) Evidence for indigenous nitrogen in sedimentary and aeolian deposits from the Curiosity rover investigations at Gale crater, Mars. Proc Natl Acad Sci DOI: 10.1073/pnas.1420932112. PMC4394254 25831544

[B136] Stern JC, Sutter B, Archer PD, *et al.* (2018) Major volatiles evolved from eolian materials in Gale Crater. Geophys Res Lett 45, 10,240–10,248.

[B137] Stroncik NA and Schmincke HU (2001) Evolution of palagonite: crystallization, chemical changes, and element budget. Geochem Geophys Geosyst 2, 2000GC000102.

[B138] Stroncik NA and Schmincke HU (2002) Palagonite—a review. Int J Earth Sci 91, 680–697.

[B139] Summers DP and Khare B (2007) Nitrogen fixation on early Mars and other terrestrial planets: experimental demonstration of abiotic fixation reactions to nitrite and nitrate. Astrobiology 7, 333–341. 17480164 10.1089/ast.2006.0032

[B140] Summers DP, Basa RCB, Khare B, *et al.* (2012) Abiotic nitrogen fixation on terrestrial planets: reduction of NO to ammonia by FeS. Astrobiology 12, 107–114. 22283408 10.1089/ast.2011.0646

[B141] Sun VZ and Milliken RE (2015) Ancient and recent clay formation on Mars as revealed from global survey of hydrous minerals in crater central peaks. J Geophys Res Atmosph 120, 2293–2332.

[B142] Svensen H, Bebout G, Kronz A, *et al.* (2008) Nitrogen geochemistry as a tracer of fluid flow in a hydrothermal vent complex in the Karoo Basin, South Africa. Geochim Cosmochim Acta 72, 4929–4947.

[B143] Swayze GA, Ehlmann BL, Milliken RE, *et al.* (2008) Discovery of the acid-sulfate mineral alunite in Terra Sirenum, Mars, using MRO CRISM: possible evidence for acid- saline lacustrine deposits? In: AGU Fall Meeting Abstracts, page A4, December.

[B144] Sweeney RE, Liu KK, and Kaplan IR (1978) Oceanic nitrogen isotopes and their uses in determining the source of sedimentary nitrogen. In: Stable Isotopes in the Earth Sciences, edited by B.W. Rob-Inson, *DSIR Bull* 220, 9–26.

[B145] Turner G, Knott SF, Ash RD, *et al.* (1997) Ar-Ar chronology of the Martian meteorite ALH84001: evidence for the timing of the early bombardment of Mars. Geochim Cosmochim Acta 61, 3835–3850. 11541217 10.1016/s0016-7037(97)00285-8

[B146] Vaniman DT, Bish DL, Ming DW, *et al.* (2014) Mineralogy of a mudstone at Yellowknife Bay, Gale Crater, Mars. Science 343, 1243480. 24324271 10.1126/science.1243480

[B147] van Zuilen M (2008) Stable isotope ratios as a biomarker on mars. Space Sci Rev 135, 221–232.

[B148] von Paris P, Grenfell JL, Rauer H, *et al.* (2013) N_2_-associated surface warming on early Mars. Planet Space Sci 82–83, 149–154.

[B149] Wadhwa M (2001) Redox state of Mars' upper mantle and crust from Eu anomalies in shergottite pyroxenes. Science 291, 1527–1530. 11222854 10.1126/science.1057594

[B150] Williams LB and Ferrell RE (1991) Ammonium substitution in illite during maturation of organic matter. Clays Clay Miner 39, 400–408.

[B151] Wong MH, Atreya SK, Mahaffy PN, *et al.* (2013) Isotopes of nitrogen on Mars: atmospheric measurements by Curiosity's mass spectrometer. Geophys Res Lett 40, 6033–6037. 26074632 10.1002/2013GL057840 PMC4459194

[B152] Zent AP, Fanale FP, and Postawko SE (1987) Carbon dioxide: adsorption on palagonite and partitioning in the martian regolith. Icarus 71, 241–249.

[B153] Zent AP, Hecht MH, Cobos DR, *et al.* (2010) Initial results from the thermal and electrical conductivity probe (TECP) on phoenix. J Geophys Res 115 (E3), 1–23.

[B154] Zerkle A and Mikhail S (2017) The geobiological nitrogen cycle: from microbes to the mantle. Geobiology 15, DOI: 10.1111/gbi.12228. PMC5412885 28158920

[B155] Zimmerman AR, Goyne KW, Chorover J, *et al.* (2004) Mineral mesopore effects on nitrogenous organic matter adsorption. Org Geochem 35, 355–375.

