# Supplementary material for: Nitrogen Incorporation in Potassic and Micro- and Meso-Porous Minerals: Potential Biogeochemical Records and Targets for Mars Sampling
Source: Astrobiology. 2022 Oct 31;22(11):1293–309. doi: 10.1089/ast.2021.0158 (PMC9618379; doi:10.1089/ast.2021.0158)
Supplement: Supplemental data [file Suppl_FigS1.docx]

*[Astrobiology]*

Supplementary Materials for

**Nitrogen Incorporation in Potassic and Micro- and Meso-porous Minerals: Potential Biogeochemical Records and Targets for Mars Sampling**

Matthew P. Nikitczuk^1^, Gray E. Bebout ^1,2^, Charles A. Geiger^3^, Tsutomu Ota^2^, Takuya Kunihiro^2^, John F. Mustard^4^, Sæmundur A. Halldórsson^5^, Eizo Nakamura^2^

^1^Department of Earth and Environmental Sciences, Lehigh University, Bethlehem, Pennsylvania, USA.

^2^Pheasant Memorial Laboratory for Geochemistry and Cosmochemistry, Institute for Planetary Materials, Okayama University, Misasa, Japan.

^3^Universität Salzburg, Fachbereich Chemie und Physik der Materialien, Salzburg, Austria.

^4^ (Department of Earth, Environmental and Planetary Sciences, Brown University, Providence, Rhode Island, USA

^5^Nordic Volcanological Center, Institute of Earth Sciences, University of Iceland, Sturlugata 7 – Askja, 101 Reykjavík, Iceland

**File Contents**

Figures S1


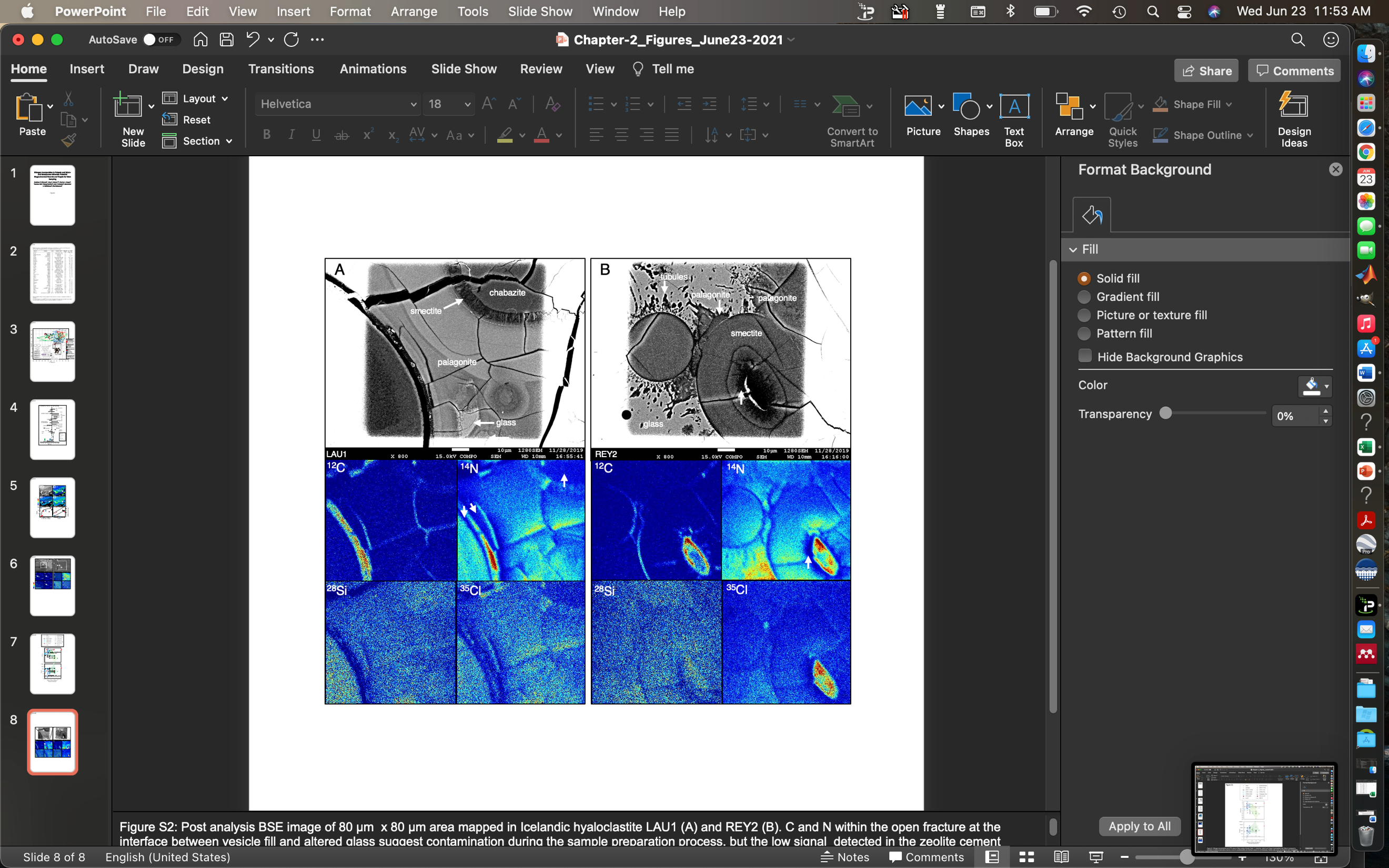


**Figure S1:** Post analysis BSE image of 80 μm x 80 μm area mapped in Icelandic hyaloclastite LAU1 (A) and REY2 (B). Carbon and N within the open fracture at the interface between vesicle fill and altered glass suggest contamination during the sample preparation process. The low signal detected in the zeolite cement (chabazite) within the vesicle at top right (A) and portions of palagonite at left (A, small white arrows in element maps) or in the inner phyllosilicate layer of layer clay coatings/infilling at the bottom right (B, white arrow) indicates that a significant quantity of N likely does not reside in these locations.
